# Molecular Arrangement and Thermal Properties of Bisamide Organogelators in the Solid State

**DOI:** 10.1021/acs.langmuir.2c02679

**Published:** 2022-11-23

**Authors:** Elmira Ghanbari, Aravind Krishnamurthy, Stephen J. Picken, Enno A. Klop, Lars J. Bannenberg, Jan van Esch

**Affiliations:** † Delft University of Technology, Delft2629 HZ, The Netherlands; ‡ Teijin Aramid Research and Innovation Centre, P.O. Box 5153, 6802 EDArnhem, The Netherlands

## Abstract

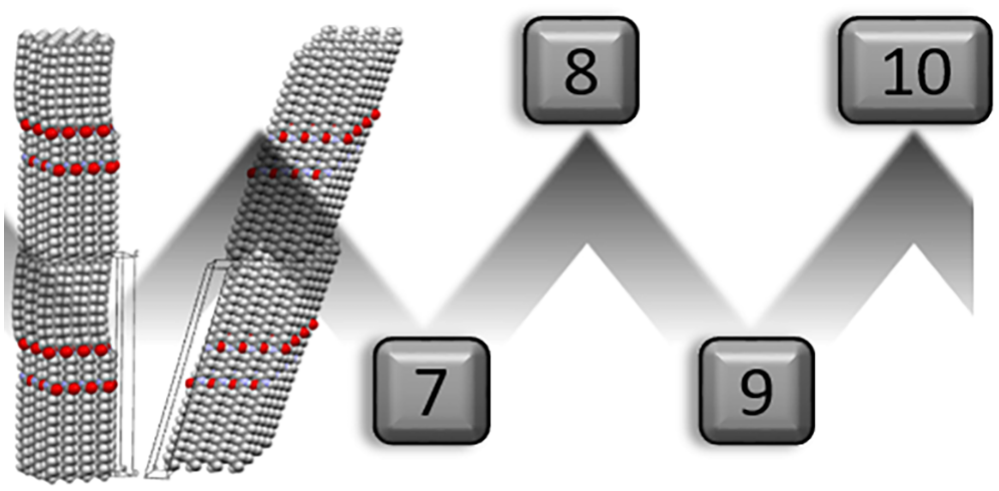

The crystal structure and phase behavior of bisamide gelators are investigated using differential scanning calorimetry (DSC), Fourier transform infrared spectroscopy, X-ray diffraction (XRD), and molecular modeling, aiming at a better understanding of bisamide gel systems. A homologous series of bisamide model compounds (*n*BAs) was prepared with the (CH_2_)_*n*_ spacer between the two amide groups, where *n* varies from 5 to 10, and with two symmetric C17 alkyl tails. With increasing spacer length, the thermal properties show a clear odd–even effect, which was characterized using our newly developed analytical model DSC_N_(T). Using XRD, all studied *n*BA compounds turn out to have a layer-like structure. The XRD patterns of the odd BA series are very similar but show marked differences compared to the XRD patterns of the even series, which in turn are very similar. The odd-membered 5BA molecules are nearly perpendicular to the stacked layers, as described by a pseudo-orthorhombic unit cell, whereas the even-membered 6BA molecules are tilted at an angle with respect to the layer normal, as described by a triclinic unit cell. In both the odd and even series, the inter-layer interaction is the van der Waals interaction. The 6BA hydrogen bonding scheme is very similar to that of Nylon 6,10 α, unlike the 5BA H bonding scheme. The packing of the C17 alkyl tails in the 5BA layers is similar to polyethylene, and unlike 6BA. The slightly higher crystalline density of 6BA (1.038 g cm^–3^) as compared to 5BA (1.018 g cm^–3^) explains the higher melting point, higher enthalpy of fusion, and the observed shift of N–H stretch bands to higher wave numbers. The structural differences observed between the odd and even BA series reflect the different structure-directing effect of parallel versus antiparallel amide hydrogen bonding motifs. These differences underlie the observed odd–even effect in the thermal properties of *n*BA compounds.

## Introduction

Low molecular weight organic gelators (LMWOGs) are low molecular mass gelling agents that are able to form supramolecular gels.^1^ They can construct gels via self-assembly, leading to the formation of micrometer-long crystals in various morphologies which entangle into an overall three-dimensional network and entrap the solvent in the network.^2−4^ The self-assembly of LMWOGs occurs through noncovalent reversible bonds such as π-stacking, hydrogen bonding, and van der Waals interactions.^5−7^

As most of the LMWOGs used as additives to increase the viscosity of liquid products were discovered fortuitously,^8−10^ there was little theoretical understanding on their mechanisms of gel formation. Finding and screening numerous compounds has directed research toward the systematic study of gelator structure, their interaction with solvents, and the correlation with the rheological properties of their final gels.^11−13^ New LMWOGs are designed rationally by stepwise modification of the molecular structure of the former gelators to optimize the rheological properties of the final gels.^9,14^

The efficiency of LMWOGs is determined by their tendency to form a stable gel at the lower gelator concentration.^15,16^ LMWOGs with long aliphatic chains or aromatic groups with a large surface combined with moieties such as ureas, urethanes, carbamates, and amides forming three-dimensional networks via strong intermolecular hydrogen bonds have shown efficient immobilization of a variety of organic solvent.^17−22^ Hanabusa et al. designed LMWOGs which gelate a wide variety of organic liquids via thermoreversible intermolecular hydrogen bonds between the N–H and C=O groups of both the amide and urethane bonds.^23^

Among these structures, amide groups are effective structural units for the formation of supramolecular gels since the formation of hydrogen bonds (H bonding) between amides is thermodynamically favored in a variety of solvents.^19^ Therefore, low molecular weight gelators which contain single^24,25^ or multiple amide groups^26−28^ can act as efficient gelators due to inducing directional intermolecular H-bonding for self-assembly.

Thermal properties such as melting of these gels were found to be associated with the strength of intermolecular H-bonding and van der Waals interactions.^29^ One of the main challenges in the rational design of these molecules has been understanding the degree of intermolecular interactions and their inherent characteristics. So far, some attempts have been made to correlate the position of amide groups in the molecular structure with the conformation of H-bonding in the network, that is, in the presence of different solvents, but still an elaborate study on the structure and properties of the solid LMWOGs as the main building units of these gel networks is required. In fact, the first step toward successful gel design and preparation is to fully characterize the structure and properties of the gelator molecules themselves.

Here, we study solely the thermal properties and molecular arrangement of bisamide LMWOGs in the solid state as they are the main active components of the gels. Understanding the structure can best be achieved by a systematic study of the structure properties relations of a homologous series of such molecules. Therefore, a series of bisamide compounds (*n*BA) were synthesized with two linear alkyl tails attached to bisamide groups with n carbon spacer length. Both odd and even linear spacers were prepared with length *n* from 5 to 10. These can be considered as simple model compounds for bisamide LMWOGs. The properties of bisamide gelators with this generic structure, that is, bisamides with the same tails, different tails, different tail lengths, or a different core structure, have been studied so far for different applications such as injectable gels, drug delivery systems, tissue engineering, and rheological modifiers in coating applications.^30−36^

Tomioka et al. studied the effect of spacer length in the bisamide structure with a series of 10-didodecanoylamides on the gelability which has shown significantly different morphologies in the gel state.^37^ The dodecanoyl tails of the molecules interact with the van der Waals forces of these bisamides. The zigzag arrangement of an odd spacer directs the two amide carbonyl groups into a parallel position, and an even spacer would appear to dictate an antiparallel alignment of amide groups. As a result of the spatial arrangement of amide groups in these two groups of bisamides, even bisamides form two pairs of hydrogen bonds with two other molecules in a single plane while bisamide molecules with odd spacer length can potentially form four independent hydrogen bonds with four other molecules not in one plane. This however would require a substantial change in the molecular conformation. By analogy, the different H-bonding patterns may cause specific self-assembly behavior for odd and even bisamides in the gel state. In fact, in the presence of appropriate organic solvents, this class of bisamide gelators can form gels with different morphologies and microstructural properties.^38,39^ The gelation process and final morphology of a series of α,ω-polymethylene bisamides, with the two amide groups bridged by even or odd numbers of spacer carbons, has been studied in a variety of solvents.^40^ It was found that the odd–even effect has a determining role on the gelability and the final properties of the gels; the bisamide gelators with even spacer length have shown the ability to gelate mesitylene. However, the longer the bridging spacer, the poorer the efficiency of gelation due to the formation of ribbon-like structures. In contrast, gelators with odd spacer length exhibited efficient immobilization of mesitylene via a fine woven fibrous network.

The present study is limited to linear symmetric bisamide gelators with a simple structure in order to understand the relation of H-bonding via amide groups with packing of these molecules in the solid state. It seems not only logical but also necessary to obtain information on the relationships between spacer length parity and the spatial arrangement of two amide groups of molecules in the solid state to be able to understand their effects on the microscopic structures in the gel state. The thermal properties and crystal structure of the molecules in the solid state have been addressed for further systematic experiments due to the fact that both the solubility and the rate of crystallization are influenced by the regularity of the H-bonding pattern.^41,42^ In fact, the analysis of structural evolution of bisamide compounds at elevated temperature is of great importance to complement our understanding of the stability of these gelators in the presence of usually poor solvents, the thermodynamics of gelation, and the intermolecular interaction, that is, dissolution or recrystallization of these compounds during the self-assembly process.

## Experimental Section

### Materials

Diamines varying in the number of methylene groups (*n*) between amine groups from 5 to 10, that is, 1,5-diaminopentane (NH_2_(CH_2_)_5_NH_2_), 1,6-diaminohexane (NH_2_(CH2)_6_NH_2_), 1,7-diaminoheptane (NH_2_(CH_2_)_7_NH_2_), 1,8-diaminooctane (NH_2_(CH_2_)_8_NH_2_), 1,9-diaminononane (NH_2_(CH_2_)_9_NH_2_), 1,10-diaminodecane (NH_2_(CH2)_10_NH_2_), and stearic acid (CH3(CH2)16COOH), Sigma-Aldrich (95–97% analytically pure); hypophosphorous acid solution; and H_3_PO_2_ (50 wt % in H_2_O), were used as received for the synthesis of bisamide molecules. Dimethyl sulfoxide-*d*_6_ (DMSO-*d*_6_) (^1^H NMR reference standard grade) was used as a solvent for the analysis by nuclear magnetic resonance (^1^H NMR).

### Synthesis and Characterization

**Figure 1:**
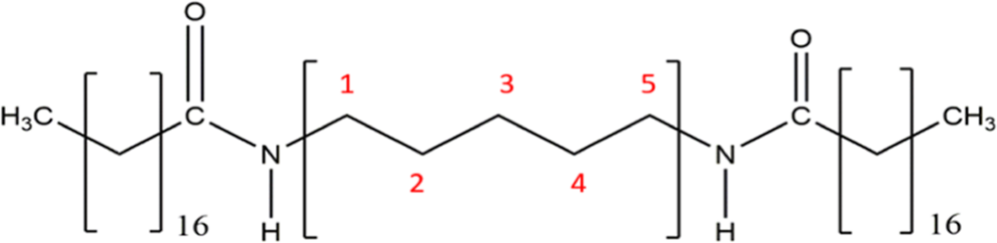
Chemical structure of the synthesized bisamides (*n*BAs) with (CH_2_)_*n*_ spacer between the amide groups (*n* = 5 in this example) and with C17 alkyl tails.

A series of bisamide compounds was produced with (CH_2_)_*n*_ spacer between the amide groups, where *n* = 5–10, and with C17 alkyl tails at each side of the amide groups. The bisamides were notated according to the number of carbon atoms in the spacer (*n*) with “BA” as the suffix for “bisamide compound”. The general chemical structure of *n*BA compounds is shown in Figure 1, using 5BA as an example.

The *n*BA series was synthesized by condensation of stearic acid and a series of diamines. Stearic acid (2 mol) and 1 mol of the appropriate *N*,*N*′-diamine were heated above their melting points and mixed via a mechanical stirrer. The synthesis was done in a neutral environment provided with N_2_ purge (99% purity). H_3_PO_2_ in negligible amounts was added as an antioxidizing agent. As a result of an amidification reaction, the bisamide compounds were synthesized in the melt state. A homogeneous yellowish white compound was produced upon cooling of the molten product.

### Proton-Nuclear Magnetic Resonance Spectroscopy

Proton NMR spectroscopy was used for all *n*BA compounds to check the successful synthesis of the compounds with the formation of amide groups. ^1^H NMR spectra were recorded on an Agilent 400-MR DD_2_ NMR spectrometer equipped with a 5 mm ONE NMR Probe. The *n*BA compounds were dissolved in DMSO-*d*_6_ (dimethyl sulfoxide *d*_6_) 5 wt % at 80 °C, and tetramethylsilane (TMS) was used as an internal reference. The resolution of the spectra was compromised due to the poor solubility of *n*BA compounds in almost any solvent.

### Differential Scanning Calorimetry

Differential scanning calorimetry (DSC) was employed to characterize the thermal behavior of *n*BA compounds. A PerkinElmer-Pyris diamond differential scanning calorimeter with two 1 g furnaces (working on the power compensation temperature null principle with accuracy < ±1% and precision < ±0.1%) was used. Nitrogen (99.99% purity) was used to purge the thermal analysis system at a rate of 50 mL/min. Temperature and heat flow calibration was done before each measurement using the heating scan of indium, a highly pure metal provided by PerkinElmer with accurately known enthalpies of fusion and melting point, Δ*H*_fusion_ = 28.47 J g^–1^ and *T*_m_^0^ = 156.4 °C, under the same condition as the to-be-measured samples. The onset of the melting transition and the area under the peak, calculated by PYRIS analytical software, were chosen, respectively, for the calibration of the melting temperature and the enthalpy of fusion.

A bisamide compound (6 ± 1 mg) was placed in a 40 μL aluminum sample pan and was weighed on a microbalance. The sample pan and a reference pan (an identical empty pan), both covered by aluminum lids, were placed in the furnaces of the DSC apparatus. Both pans were heated from room temperature to at least 30 °C above and below the temperature range of interest. Isothermal melting was followed by a fixed cooling cycle preceding a second heating cycle, all scans at the rate of 10 K min^–1^. In fact, the identical method was performed in order to eliminate the thermal history of the compounds which might have been caused during the compound synthesis and sample preparation processes. Therefore, the data recorded from the second heating cycle can be used for further analysis to obtain information solely on the effect of different spacer lengths of *n*BA on their thermal properties. Prior to the data collection for the analysis, the heat flow of the raw data (mW) was normalized per weight of the sample (mg), resulting in “normalized heat flows” (W/g). The normalized data were transferred from the PerkinElmer computer into ASCII format. The data visualization was done by Python and the endothermic peaks were plotted in upward direction in all graphs.

### DSC_N_(T) Analytical Model and Curve Fitting

Based on the thermodynamics of melting phenomena, a DSC analytical model, DSC_N_(T), has been developed, which fits the DSC experimental traces by capturing the peak shape.^43^ The model allows fitting of the DSC peaks taking an assumed Arrhenius crystal size distribution together with instrumental and sample-related peak broadening into account and yields a much more accurate determination of the equilibrium melting point, enthalpy of fusion, and change in heat capacity of *n*BA compounds. The nonlinear curve fitting of DSC_N_(T) to the experimental DSC traces has been done using the Python 3 programming language. The nonlinear least squares (NLLSs) function from the scipy.optimize.curve_fit module was used, which takes the independent variable and the function parameters and optimizes the parameters within defined lower and upper bounds to minimize the sum of squares of nonlinear functions. The curve fitting consisted of the entire temperature range on the *x*-axis, which is broad enough to cover the peak region and to precisely determine the baseline on the tails at both sides of the peak minimum. This method improves the reproducibility of the fitting process and the precision of the fitted parameters.

To extract accurate information from DSC measurements, at least three *n*BA samples with nearly identical weight were measured under the same condition. The standard deviation of the melting temperature, enthalpy of fusion, and heat capacity change were obtained by fitting the analytical model DSC_N_(T) to the three sets of raw data which contains the experimental error along with the fitting procedure error. The fitting deviation for each parameter was obtained from the residuals of NLLS. The reported error margins of the fit parameters in the table are the residuals of NLLS rounded to two digits.

### Powder X-ray Diffraction and Molecular Modeling

X-ray diffraction (XRD) combined with molecular modeling was used to obtain information on the crystal structure of *n*BA compounds. XRD patterns were recorded at room temperature with a Bruker D8 ADVANCE ECO diffractometer in Bragg–Brentano geometry, equipped with a Cu X-ray source (*K*_α1_ = 1.54060 Å and *K*_α2_ = 1.54439 Å) and a LYNXEYE-XE-T position sensitive detector. A knife-edge has been used to reduce the background due to the scattering of the primary beam. The patterns were recorded from 0.6 to 50°(2θ) with a step size of 0.01° and a measuring time of 0.5 s/step. The Cerius2 software package (version 4.2, from Accelrys, now owned by Biovia) was used to build the crystal structure models, employing the Compass force field. Simulated XRD patterns were calculated using the Cerius2 diffraction module and the GSAS-2 software system.^44^ The Lorentz and polarization factors were included in the calculated reflection intensities. The crystallite sizes were chosen to match the observed diffraction patterns.

Temperature-dependent XRD measurements were performed with a Bruker D8 ADVANCE in reflection mode equipped with a Co X-ray source (*K*_α1_ = 1.78897 Å and *K*_α2_ = 1.79285 Å) and a LynxEYE-XE position sensitive detector. Variable divergence slits were used to achieve a constant footprint on the sample, which itself is located inside an Anton Paar XRK900. The sample was loaded into a Macor ceramic sample holder designed to minimize systematic errors on the data (i.e., shift in 2θ position) caused by the thermal expansion of the sample holder resulting in a different height of the sample. The measurements were performed by stepwise increasing the temperature at a rate of 10 K min^–1^.

### Fourier Transform Infrared Spectroscopy

To study the supramolecular interaction of bisamide compounds, Fourier transform infrared (FTIR) spectroscopy with an attenuated total reflection (ATR) method was carried out. ATR–FTIR spectra were recorded on a FTIR spectrophotometer (Nicolet 6700 from Thermo Fisher Inc., USA) equipped with an ATR attachment with a diamond crystal. The appropriate amount of *n*BA compound was placed on the crystal, and then its FTIR spectrum was recorded. The resolution of the spectra was 2 cm^–1^. The final spectra were acquired as the average of 32 scans.

## Results and Discussion

### Synthesis of Bisamide Gelators

Bisamide compounds *n*BA with spacer length *n* = 5–10 and with C17 alkyl tails at both amide groups were synthesized. The ^1^H NMR spectra show that the amidification reaction has occurred, and the *n*BA compounds were produced in high yield (Table S1). The ^1^H NMR spectra of all *n*BA compounds were comparable with those reported in the literature (Figure S1).

### Thermal Analysis

**Figure 2:**
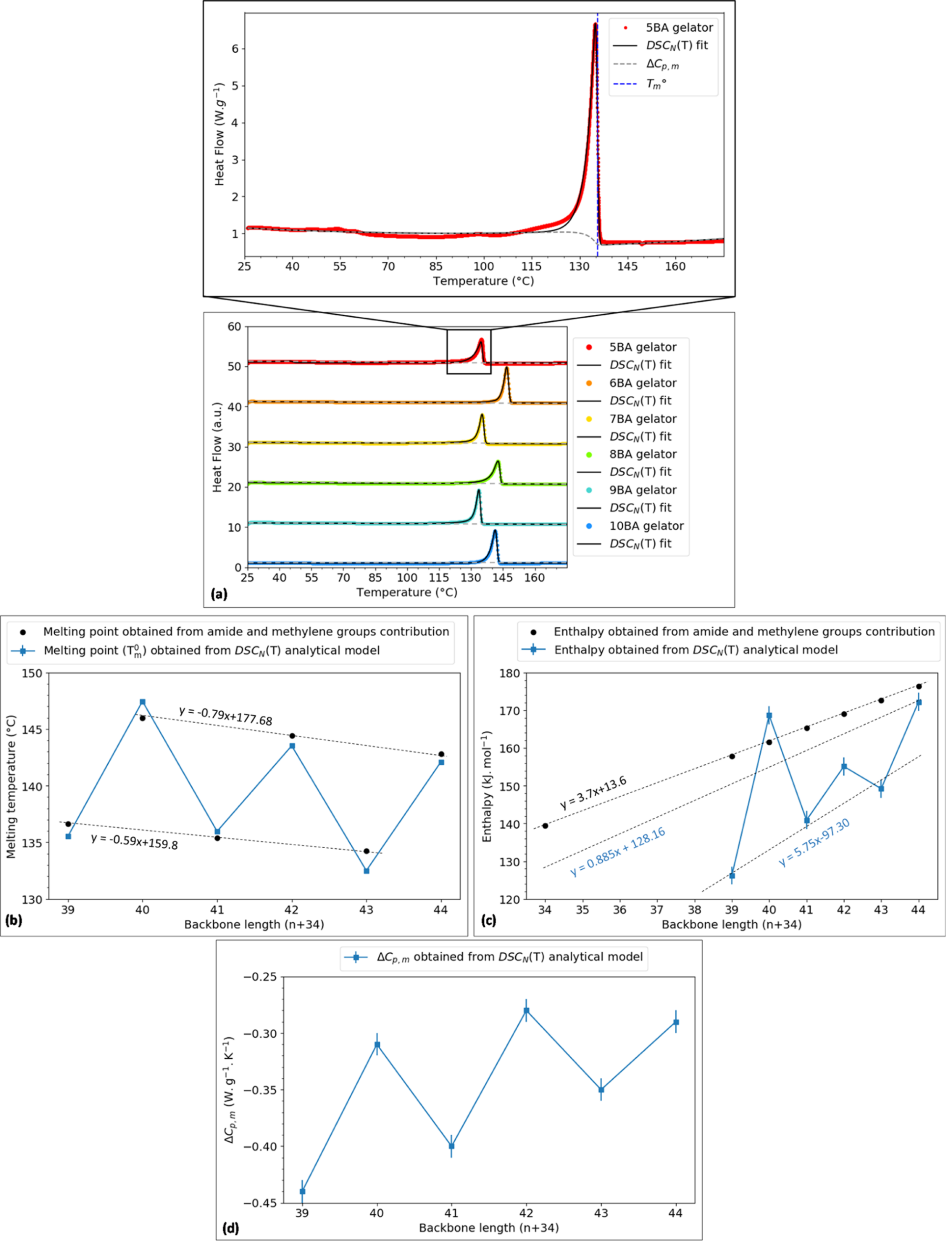
(a) DSC_N_(T) function fitted to the second heating traces (endo up) representing the melting transition of *n*BA compounds measured at 10 K min^–1^ after calibration at the onset for the given sample weight and scan rate, (b–d) thermal properties of BA compounds showing the odd–even effect: (b) melting temperatures (*T*_m_^0^) obtained from the DSC_N_(T) analytical model and calculated using tentative values of methylene and amide group contributions, (c) enthalpy of fusion obtained from the DSC_N_(T) analytical model and calculated using tentative values of methylene and amide group contributions, and (d) change in heat capacity obtained from the DSC_N_(T) analytical model (the errors of change in enthalpy and heat capacity are the experimental errors obtained from measurements on three samples analyzed by DSC_N_(T), and for the melting points obtained from DSC_N_(T), the errors are the fitting residuals calculated using DSC_N_(T), SD = 0.00 °C).

The effect of spacer length on the phase behavior of *n*BA compounds was studied with DSC. Figure 2a shows the second heating DSC traces of *n*BA compounds scanned from 25 to 180 °C. All *n*BA compounds exhibit transition peaks between 120 and 150 °C. The melting points of the solid-state bisamide gelators with even spacer lengths and C17 alkyl tails have been reported to be around 140 °C.^45,46^ Therefore, the transition peaks in heat flow versus temperature diagram (Figure 2a) are assigned to the melting transitions of the *n*BA compounds.

The thermodynamic melting point of a fully crystalline and 100% pure compound is in theory an infinitely sharp peak in a DSC thermogram.^47^ However, in real *n*BA samples, a distribution of crystal sizes exists, which causes an asymmetric shape for the melting transitions in the DSC trace (Figure 2a). Other factors broadening the peak include limited instrumental resolution and thermal gradients in the sample. Moreover, the presence of impurities in the samples can cause additional broadening. As a result of these effects, the DSC signal would turn into a symmetric Gaussian distribution. The onset of the melting peak is usually considered as the onset of the melting transition where the extrapolated baseline and the tangent of the rising edge intersect; we find, however, that this method of analysis should be changed in the case of severely asymmetric peaks, as is the case for *n*BA compounds.

In case of a broad, crystal size-induced, asymmetric melting point depression, the convolution of a truncated Arrhenius base function with Gaussian broadening provides a convenient and rather versatile asymmetric melting peak function. Equation 1 gives the analytical DSC_N_(T) model that we derived, which yields the equilibrium melting temperature (*T*_m_^0^), the enthalpy of fusion (Δ*H*), and the change in heat capacity prior and after the transition (Δ*C*_p,m_) as described by eq 2.^43^ The mathematical terms and physical attributions of the parameters of DSC_N_(T) are summarized in Table 1.
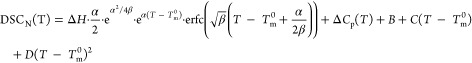
1
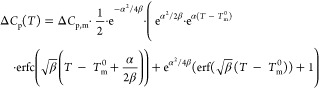
2

**Table 1:** Variables, Functions, and Parameters of DSC_N_(T)

parameter	power units	thermodynamic units	physical attributions and mathematical terms
*T*_m_^0^	°C	K	the equilibrium melting point of the phase transition
*B*	W g^–1^	J g^–1^ K^–1^	baseline offset
*C*	W g^–1^ K^–1^	J g^–1^ s^–1^	linear baseline slope
*D*	W g^–1^ K^–2^	J g^–1^ s^–1^ K^–2^	second-order baseline curvature
α	K^–1^	K^–1^	strength of the linearized Arrhenius function (α = *E*_a_/(*R*·(*T*_m_^0^)^2^), describing the crystal size distribution, roughly proportional to the steepness of the rising edge of the peak
β	K^–2^	K^–2^	the parameter in relation to the Gaussian distribution of the peak ( 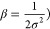 , describing the peak broadening in the declining edge
Δ*C*_p_,m	W g^–1^ K^–1^	J K^–1^	the difference between the heat capacity of the solid and liquid state
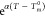			the Arrhenius function determining the rising edge of the curve
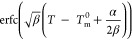			Erfc, the complementary error function, describes the falling edge of the peak as it returns to the baseline
Δ*H*	W g^–1^	J g^–1^	the coefficient of DSC_N_(T) function representing the change in enthalpy associated with the phase transition
*R*^2^			the statistical measure for the goodness of fit in a regression function that determines the amount of variance in the dependent variable that can be explained by the independent variable

Figure 2a shows that the DSC analytical model fits the second experimental heating traces of all *n*BA compounds remarkably well (*R*^2^ > 0.99) (Table S2). Shown by the dashed blue line in the inset for 5BA as an example, the equilibrium melting point (*T*_m_^0^) obtained from the DSC_N_(T) model is at a different location than the onset of the peak. In fact, it occurs on the trailing edge of the DSC peak.

Calculated based on DSC_N_(T), Figure 2b manifests the odd–even alternation of the equilibrium melting temperature (*T*_m_^0^), enthalpy of fusion (Δ*H*) (Figure 2c), and change in heat capacity prior to and after transition (Δ*C*_p,m_) (Figure 2d) versus the spacer length of *n*BA compounds. We find that *T*_m_^0^, Δ*H*, and Δ*C*_p,m_ of even *n*BAs are higher than those of the odd *n*BAs, where the not too dramatic difference refers to the similarities in crystal structure and molecular arrangement that will be discussed in the XRD analysis section.

Figure 2b,c compares the melting temperature and enthalpy of fusion of *n*BA compounds calculated from the DSC_N_(T) analytical model and from the tentative values of enthalpy and entropy change of melting of methylene (CH_2_) and amide groups (CONH) suggested by van Krevelen et al.^48^ All bisamides consist of two amide groups and *n* + 34 methylene groups (*n* as spacer length and 17 in each tail) in their structures. The enthalpy of fusion of the bisamide increases upon increasing the spacer length since the length of the entire backbone increases, which reinforces the van der Waals interaction among these molecules. The calculation of enthalpy of fusion from the enthalpy per CONH and CH_2_ results in average values between odd and even converging to the enthalpy of polyethylene (PE) where CH_2_ groups are the only moieties in the backbone. Thus, the van der Waals force is the sole force controlling the interactions between these “infinite” bisamide molecules; however, the alternation in DSC_N_(T) values indicates that the H-bonding via amide moieties contributes to the enthalpy, which is known as the odd–even effect. The enthalpy of fusion for the contribution of the CONH group for even bisamides and odd bisamides is 7.8 and 5.8 kJ mol^–1^, respectively, indicating that the odd–even alternation in properties results from the packing of the molecules in the solid state.^49^

### Crystal Structure

#### Room-Temperature XRD

To investigate the molecular arrangement and crystal structure of the BA compounds in the solid state, XRD patterns of the synthesized *n*BA compounds were recorded at ambient temperatures. Figure 7 displays the XRD patterns of the odd BAs and Figure 8 displays those of the even BAs (see also Figure S2). The 5BA and 6BA crystal structures were investigated in detail, as they represent odd and even BAs, respectively.

#### 5BA (Odd *n*BAs)

An initial model for the crystal structure of 5BA was built and energy-minimized with free unit cell parameters using the Cerius2 software. Calculation of the XRD pattern based on this model allows indexing of the observed XRD pattern. Subsequently, unit cell refinement was carried out via least-squares refinement using a suitable set of observed 2θ peak positions (Figure S3). The final unit cell for the 5BA model is triclinic; parameters are given in Table 2. The calculated 2θ peak positions based on the refined unit cell are very close (within 0.8%) to the observed values (Table 3), implying successful unit cell determination and refinement.

**Table 2:** Refined Unit Cell Parameters of 5BA and 6BA, Respectively, Compared to PE and Nylon 6,10 α (from the Literature^50,51^); ρ_c_ is the Crystalline Density

compound	unit cell	*a* (Å)	*b* (Å)	*c* (Å)	α (deg)	β (deg)	γ (deg)	*Z*	symmetry	ρ_c_ g cm^–3^
5BA	triclinic (pseudo-orthorhombic)	8.55	4.36	55.58	90.0	92.1	91.9	2	*P*1	1.018
PE	orthorhombic	7.39	4.30	2.54	90	90	90			0.96
6BA	triclinic	5.02	5.29	57.46	48.7	76.8	65.2	1	*P*1̅	1.038
Nylon 6,10 α	triclinic	4.95	5.40	22.40	49.0	76.5	63.5			1.07–1.09

**Table 3:**
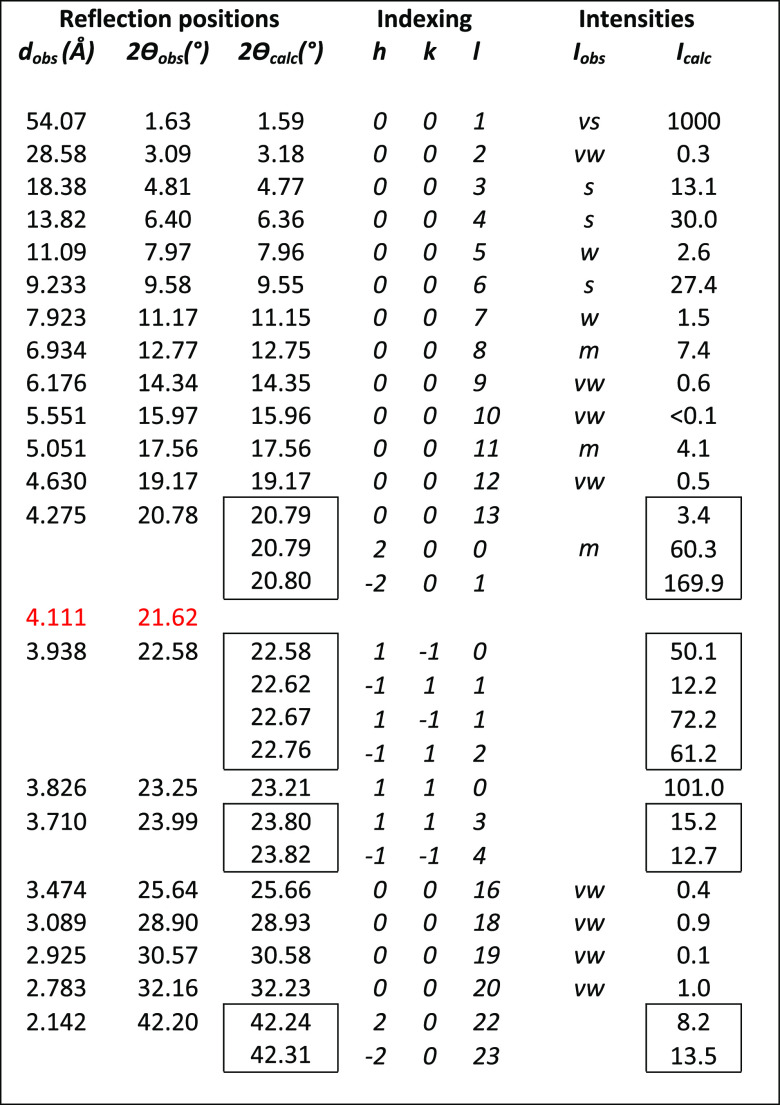
Observed versus Calculated Peak Positions and Intensities for the Sheet-like 5BA Model, Assuming Random Orientation^a^

a Intensities of 00*l* reflections are marked as very weak (vw), weak (w), medium (m), strong (s), or very strong (vs); the other reflections are not on the same intensity scale due to preferred orientation. The reflection indicated in red is not explained by the model and may point to polymorphy.

The refined unit cell was used as constraint in subsequent COMPASS energy minimizations to obtain the final 5BA crystal structure models. Two different models (see Figure 6) match the data, as will be discussed in more detail later: a sheet-like model and a bi-directionally hydrogen-bonded model (2D H-bonding), sharing the same unit cell dimensions. Atomic coordinates are available in Table S3 and S4. The sheet-like model was used to produce the calculated intensities listed in Table 3, assuming random crystallite orientation in the powder. The 00*l* intensities are reproduced very well, including the near absence of the 00*l* reflections marked as very weak (vw) or weak (w). The presence of higher order 00*l* reflections up to *l* = 20 at 2θ = 32.16° indicates an exceptionally regular layer spacing. The observed non-00*l* reflections show severe overlap, unlike the 00*l* reflections. They are weaker than calculated, indicating preferred orientation.

**Figure 3:**
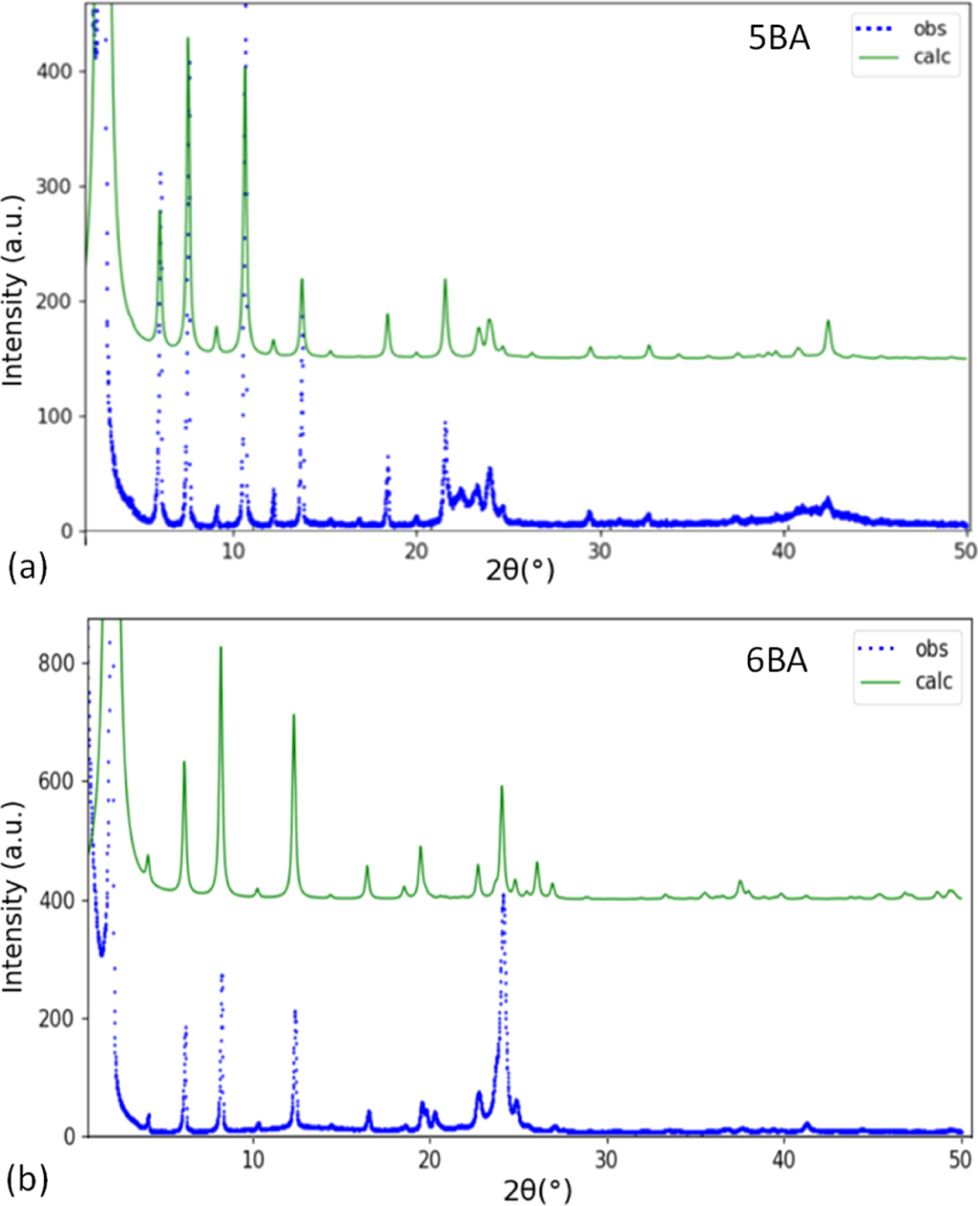
Calculated XRD pattern (upper curve) compared to the observed XRD pattern (lower curve) for (a) sheet-like model of 5BA and (b) 6BA. In the calculated patterns, preferred orientation is taken into account.

Figure 3a presents the calculated XRD pattern based on the sheet-like crystal structure model of 5BA, together with the observed XRD pattern of 5BA. The simulated pattern was calculated using the GSAS-2 software package,^44^ taking preferred orientation into account via the March–Dollase model with unique reflection 001. The simulated pattern (not shown) for the bi-directional H-bonding model of 5BA is very similar to that of the sheet-like model. Figure 3a shows that the simulated 5BA pattern matches the observed pattern very well; even the weak peaks in the high angle region are nicely reproduced. The observed pattern shows one extra peak (indicated in red in Table 3), which may be due to a different polymorph.

**Figure 4:**
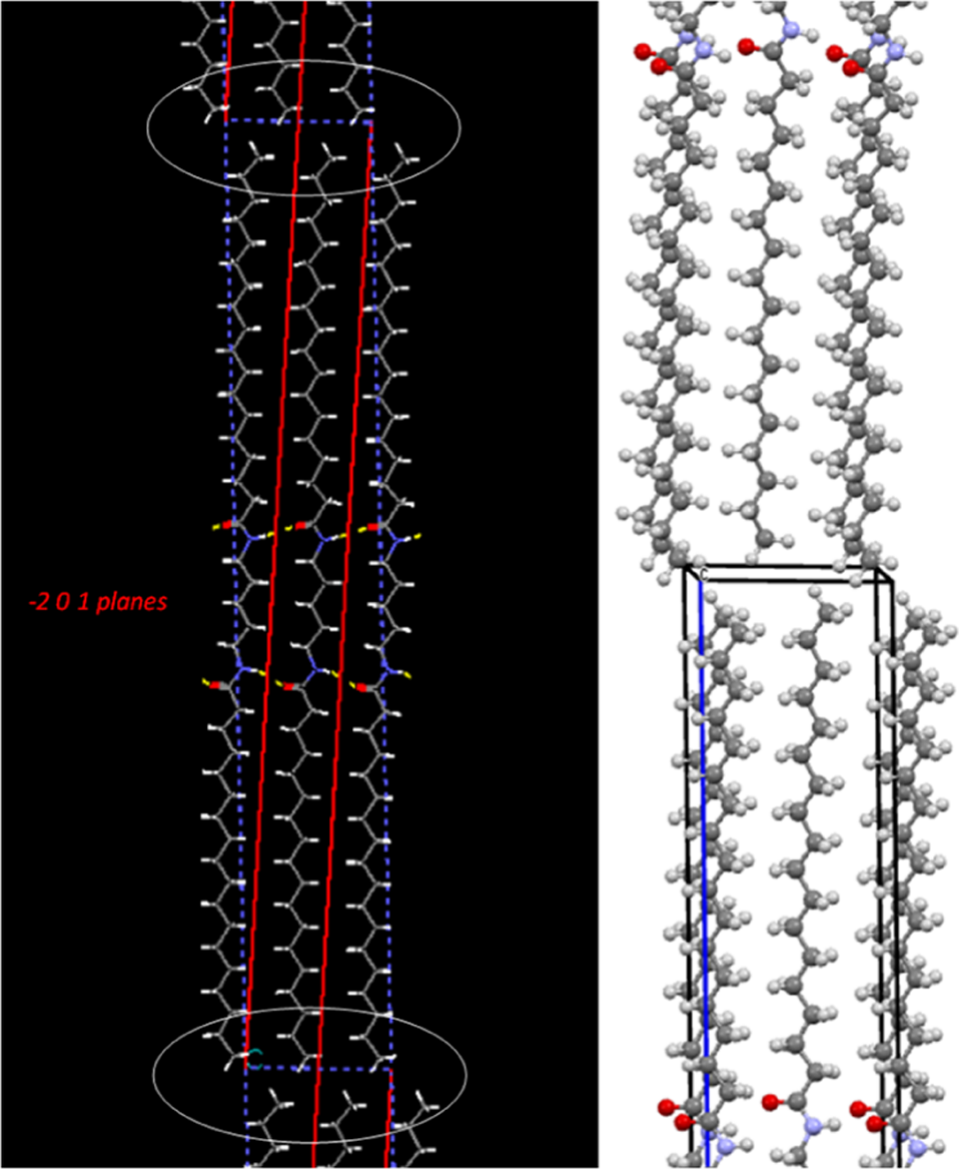
Molecular axis tilting of 5BA, tilting of molecules is in the *ac*-plane (blue dashed lines), and white ellipses highlight the methyl groups which avoid each other to minimize steric hindrance. The red lines indicate the (−2 0 1) planes.

The 5BA crystal structure has a triclinic unit cell, which is pseudo-orthorhombic since the angles are very close to 90° (Table 2). The 5BA molecular axes (in both models) are not completely parallel to the *c*-axis but show pronounced tilting in the *ac*-plane, referred to as axis tilting. The tilted axes are reasonably parallel to the (−2 0 1) planes (indicated in red), as is clear from Figure 4. This contributes to the high intensity of the −2 0 1 reflection listed in Table 3.

**Figure 5:**
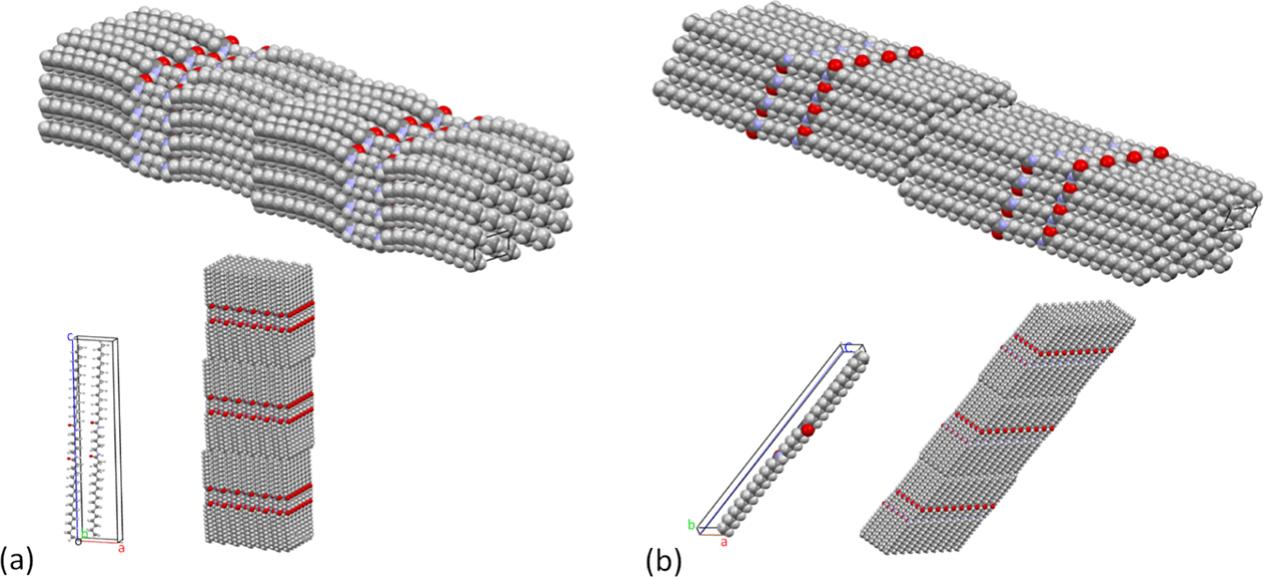
Crystal structure of 5BA and 6BA showing layer stacking: (a) long axes of the 5BA molecules are nearly perpendicular to the stacked layers as described by the pseudo-orthorhombic unit cell (with two molecules per cell), (b) 6BA molecules are at an angle to the stacked layers as described by the triclinic unit cell. The 6BA unit cell has one molecule per cell and shows the molecular axis tilting effect, just like 5BA. Sheet-like H-bonding between 6BA molecules occurs along the (010) planes in the stacked layers.

Zooming out from a single unit cell to a 5BA crystallite, it is clear that the 5BA gelator molecules are packed in layers with the long axis of the molecules nearly perpendicular to the layers (Figure 5a). The interaction between the layers is governed by van der Waals forces. In fact, the above-mentioned tilting effect allows optimal packing of the methyl groups and adequately takes into account the influence of neighboring layers, or in other words the typical layer stacking. The layer stacking may, however, be influenced by a typical form of layer stacking polymorphy often seen in layer-like materials, that is, by the particular polytype exhibited by the 5BA crystallites. The presented models are monolayer models. Two-layer polytype models can be constructed simply by doubling the unit cell along the *c*-axis and (e.g.) introducing inversion symmetry. This results in *Z* = 4, *P*1̅, models with the same unit cell parameters as the monolayer models except for the *c*-axis, which is twice long. Although it is good to be aware of the possibility of polytype polymorphy, such models are not explored here since they are out of the scope of the present investigation.

**Figure 6:**
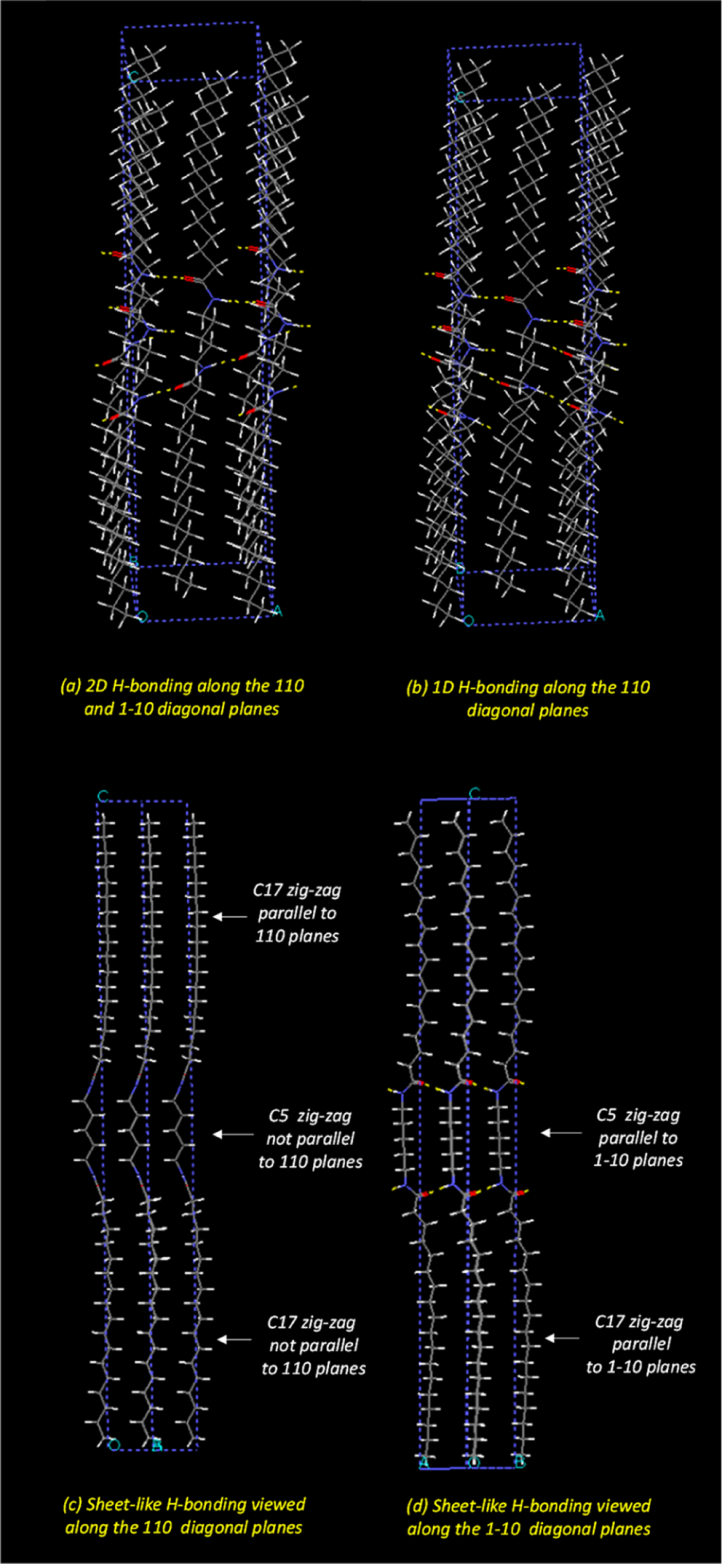
5BA structural models: (a) 2D H-bonding in the bi-directionally hydrogen-bonded structure and (b) 1D H-bonding in the sheet-like structure, viewed along two different diagonal directions: (c) along the (110) diagonal planes and (d) along the (1 −1 0) diagonal planes.

The 5BA structural models show 1D and 2D H-bonding as schematically illustrated in Figure 6a,b, respectively. Clearly, this intra-layer H-bonding interaction is stronger than the van der Waals interaction between the stacked layers (i.e., the inter-layer interaction). The molecules are hydrogen-bonded along the (110) diagonal planes for the sheet-like structure and along both (110) and (1–10), for the bi-directionally hydrogen-bonded structure. The sheet-like structure is lower in energy by 8.37 kJ mol^–1^. The actual structure may be a mixture of both hydrogen bonding schemes.

Figure 6c,d shows the sheet-like 5BA model viewed along two different diagonal directions, that is, along the (110) and (1 −1 0) crystallographic planes. The backbone of the two C17 alkyl tails (i.e., the “zigzag”) is positioned in the crystal structure in such a way that the backbones of the two tails are in the two different diagonal planes, instead of only in one plane. This C17 alkyl tail rotation effect is attributed to the odd C5 spacer, which induces a rotation of the alkyl tail from one diagonal plane in the pseudo-orthorhombic unit cell to the other. The C5 alkyl spacer backbone is parallel to the (1 −1 0) planes. Compared to the polymer chain packing in PE, the packing of the C17 alkyl tails in the *ab*-plane of 5BA is similar since they both have a pseudo-orthorhombic *Z* = 2 projection cell (exactly orthorhombic for PE as shown in Table 2). However, the molecular packing of 5BA is more dense than the polymer chain packing in PE, especially along the *a*-axis. Moreover, the alkyl tails do not have a herringbone-like packing as in PE.^50^

**Figure 7:**
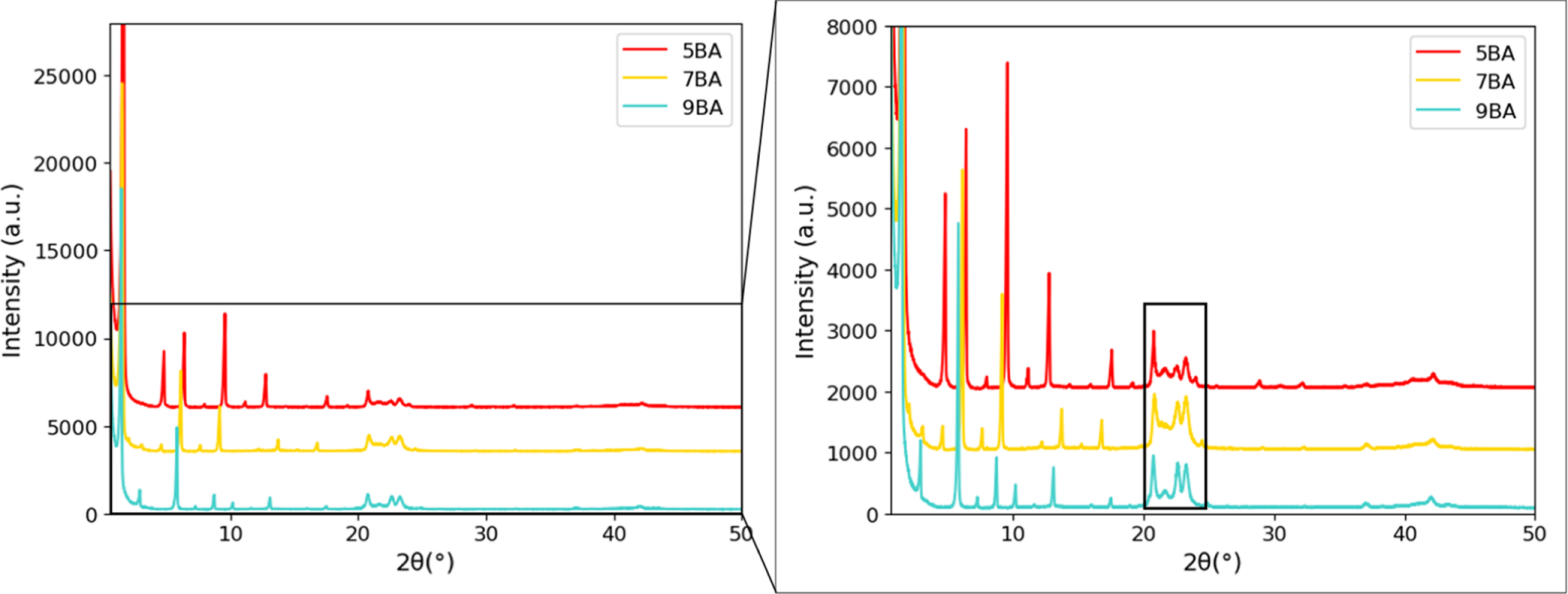
Observed XRD patterns of odd *n*BA gelators, including zoomed-in figure. The 00*l* reflections shift to lower angles with increasing spacer length. The black box emphasizes reflections in the 20–25°(2θ) range, which are hardly influenced by the spacer length.

The observed XRD patterns of the three odd BAs are shown in Figure 7. The characteristic lamellar 00*l* reflections observed for 5BA (Table 3) are observed for 7BA and 9BA as well, indicating a highly defined layer spacing. The shift to lower angles with increasing spacer length from 5BA to 9BA is due to an increasing *c*-axis length. The reflections in the 20–25°(2θ) range are very similar for all odd gelators, implying that the side-by-side packing of 7BA and 9BA molecules is very similar to that found for 5BA.

#### 6BA (Even *n*BAs)

An initial crystal structure model for 6BA was built using molecular modeling and diffraction pattern matching. The model allows indexing of a subset of observed XRD peak positions (Figure S4), which was subsequently employed in a least-squares refinement of the cell parameters. A zero-shift correction was applied, which refined to −0.061*°* (2θ). The refined unit cell parameters are listed in Table 2. COMPASS energy minimization using the refined unit cell parameters as constraints produces the final model of which the atomic coordinates are listed in Table S5. The simulated XRD pattern based on the model, taking into account the preferred orientation, shows a good match with the observed XRD pattern (Figure 3b). The calculated 2θ peak positions based on the refined unit cell are very close (within 0.8%) to the observed values (Table 4), implying successful unit cell determination and refinement. The refined model was used to produce the calculated intensities listed in Table 4, assuming random orientation.

**Table 4:**
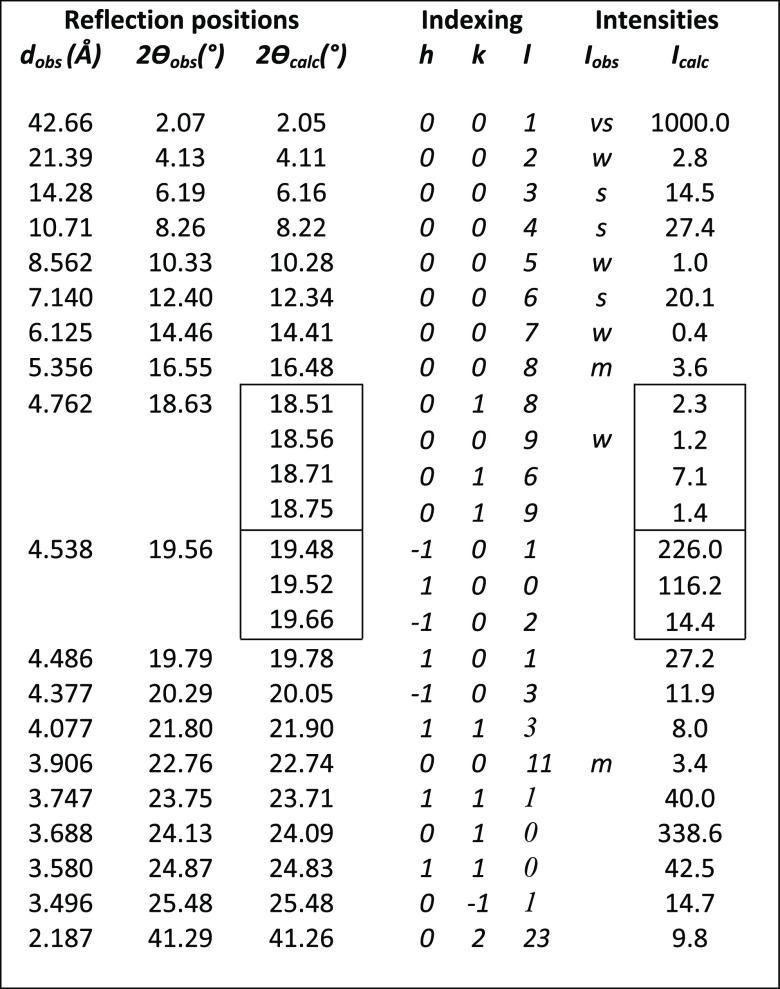
Observed versus Calculated Peak Positions and Intensities Based on the 6BA Model, Assuming Random Orientation^a^

a The intensities of 00*l* reflections are marked as very weak (vw), weak (w), medium (m), strong (s), or very strong (vs); the other reflections are not on the same intensity scale due to preferred orientation.

The refined model is a monolayer model (Figure 5b), which exhibits similar layer stacking as found for 5BA, again with the van der Waals interaction as an inter-layer interaction. Also similar to 5BA, 6BA exhibits a molecular axis tilting effect; that is, the molecular axes are tilted in the *ac*-plane, with respect to the *c*-axis, which ensures minimal steric hindrance between the methyl groups. However, 6BA has a triclinic unit cell with one molecule per cell (*Z* = 1), very different from the pseudo-orthorhombic unit cell of 5BA with *Z* = 2 (Table 2). The inversion symmetry of the 6BA molecules translates to *P*1̅ space group symmetry, unlike 5BA, where the molecules do not have inversion symmetry and the space group symmetry is P1. There is no alkyl tail rotation effect in 6BA unlike in 5BA, where the odd C5 spacer induces alkyl tail rotation. 6BA has a slightly higher crystalline density (1.038 g cm^–3^) as compared to 5BA (1.018 g cm^–3^). The long axes of the 6BA molecules are at an angle to the layers, as described by the triclinic unit cell.

Comparing the molecular packing of 6BA with that of nylons, the 6BA cell parameters are very close to those of Nylon 6,10 α,^51^ except for the *c*-axis, which is much longer for 6BA (Table 2). The 6BA molecules exhibit sheet-like H-bonding (Figure S6) in each stacked layer along the (010) planes, very similar to Nylon 6,10 α. In fact, the molecular backbones of the C6 spacer and C17 tails are in the plane of the hydrogen-bonded sheets, which is similar to Nylon 6,10 α.

**Figure 8:**
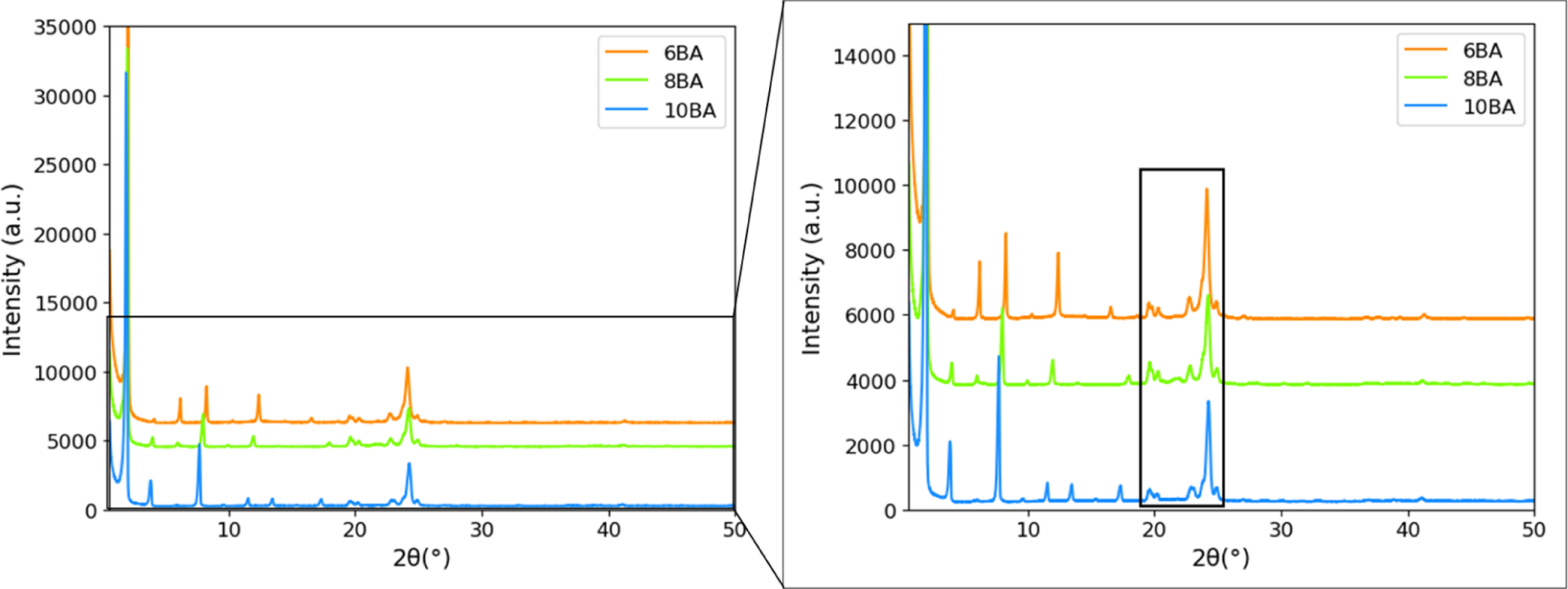
Observed XRD patterns of even bisamide gelators, including zoomed-in figure. The 00*l* reflections shift to lower angles with increasing spacer length. The black box emphasizes the reflections in the 19–25° (2θ) range, which are hardly influenced by the spacer length.

The XRD patterns of the even BAs displayed in Figure 8 not only show 00*l* reflections for 6BA but also for 8BA and 10BA. The *c*-axis length increases with increasing spacer length, which leads to a low-angle shift of the 00*l* peaks. The reflections in the 19–25°(2θ) range are very similar for the even gelators, implying that the side-by-side packing of 8BA and 10BA is very similar to that of 6BA.

### High-Temperature XRD

**Figure 9:**
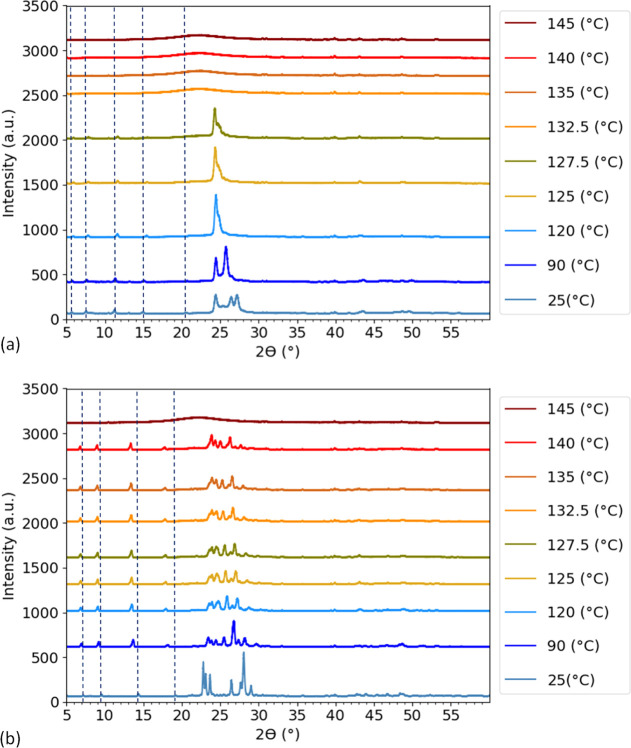
XRD patterns of (a) 5BA and (b) 6BA at different temperatures showing that melting of 5BA and 6BA has occurred around 132.5 and 145 °C, respectively; dashed lines show the shifting 00*l* reflections.

To investigate the structural evolution of the *n*BA compounds and their phase behavior at high temperature, their XRD patterns at elevated temperature were recorded. Inspecting the XRD patterns (Figure 9a,b) at elevated temperatures, it is clear that the crystalline peaks of these compounds disappear and turn into a very broad peak around 132.5 and 145 °C for 5BA and 6BA, respectively. These broad peaks are due to the amorphous structure of the liquid state formed upon melting, which is in agreement with the melting transitions obtained from DSC_N_(T) (*T*_m_^0^ = 135.52 °C for 5BA and *T*_m_^0^ = 147.46 °C for 6BA). The melting transition detected by XRD differs from DSC measurement by a few degrees, which is due to the different measurement equipment and conditions.

The XRD patterns in Figure 9 were recorded with Co radiation, unlike the XRD patterns in Figures 3, 7, and 8, which were recorded with Cu radiation. Hence, the patterns in Figure 9 are shifted to higher angles compared to the measurements with Cu radiation due to the different wavelengths (Bragg’s law). With increasing temperature, the 00*l* peaks slightly shift to higher angles (Figure 9a), which is in agreement with thermal motion, causing a slight contraction of the molecular axes. The reflections in the 25–28°(2θ) range start to merge with increasing temperature, suggesting a gradual change to a somewhat higher symmetric structure, which may be a disorder phase.

The structural evolution of 6BA is quite different from that of 5BA. While for 5BA the 00*l* reflections indicate a small contraction of the molecular length, the 00*l* reflections for 6BA show a marked shift to lower angles (Figure 9b), indicating that the layer spacing increases. This points to a change in lattice angles, leading to a smaller angle of the molecular axes with respect to the normal to the stacked layers. At the same time, the reflections in the 23–30° (2θ) range change considerably, but they do not gradually merge as in the case of 5BA. These changes point to a solid-state transition to a very different high-temperature structure, occurring in the temperature range below 120 °C. Remarkably, this structure seems to be quite robust since it remains intact up to melting.

### Fourier Transform Infrared Spectroscopy

**Figure 10:**
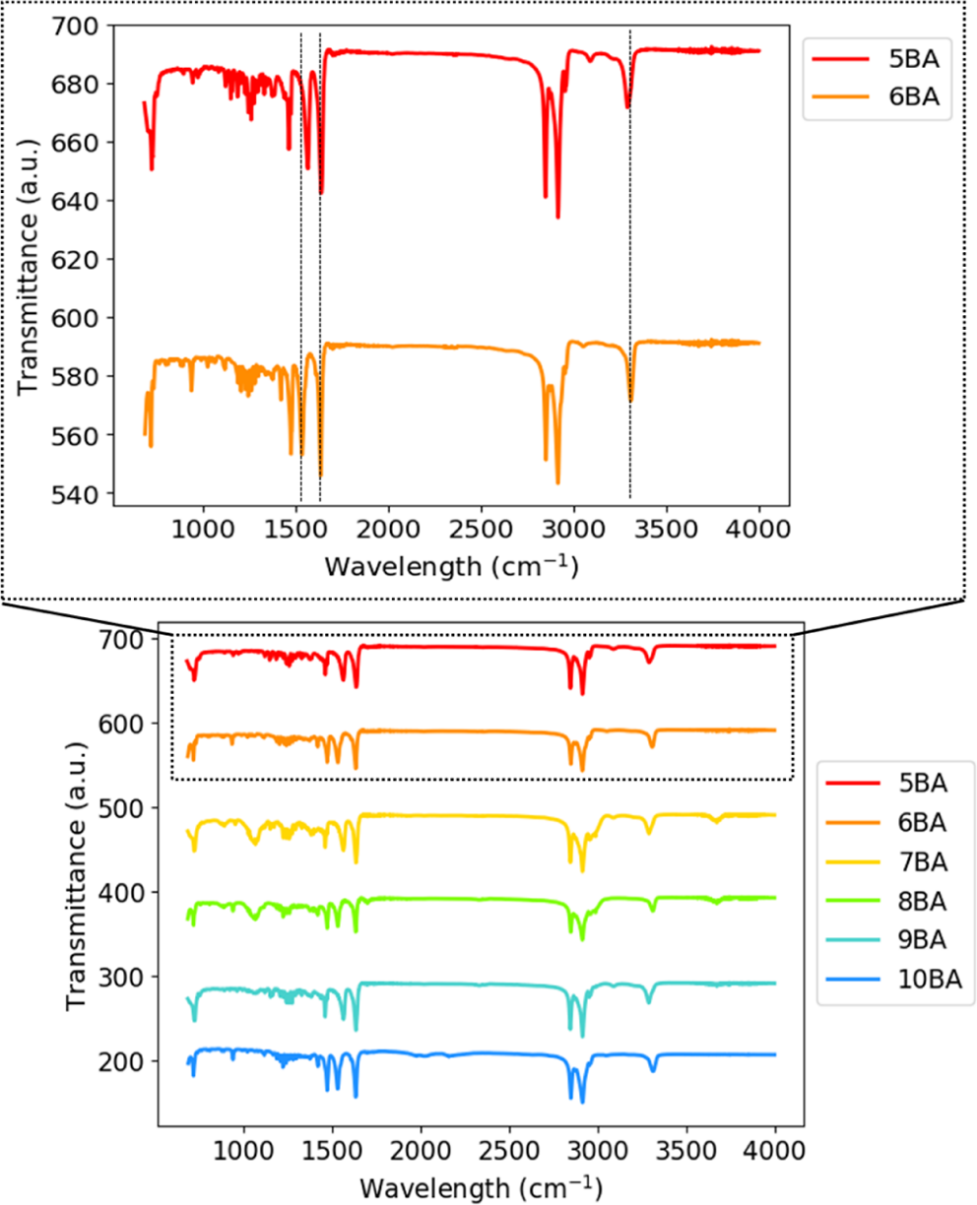
FTIR spectra of *n*BA compounds.

FTIR spectroscopy was used to probe the H-bonding interactions where several absorption bands in the infrared region are related to the H-bonding pattern. The ATR-FTIR spectra of the *n*BA compounds in the solid state are displayed in Figure 10.

The symmetric and asymmetric stretching peaks of −CH_2_ and −CH_3_ groups were observed around 2844 and 2918 cm^–1^, respectively. As expected, the relative intensity of the −CH_2_ peak increases with increasing spacer length. N–H (stretch), C=O (amide I), and N–H bending (amide II) are the characteristic bands involved in the H-bonding between *n*BA molecules; thus, they are sensitive to changes in the H-bonding pattern. The C=O (amide I) band was found to be sensitive to molecular arrangement since an alternating shift was detected between the spectra of the odd and even series (Table 5), for instance, the C=O (amide I) band shifts from 1635 cm^–1^ for 5BA to 1633 cm^–1^ for 6BA as can be seen most clearly from the inset on top of Figure 10.

**Table 5:** Positions of FTIR Bands for Odd and Even Bisamide Compounds

compound	N–H stretch (cm^–1^)	C=O (amide I) (cm^–1^)	N–H bending (amide II) (cm^–1^)
5BA	3291	1635	1563
6BA	3309	1633	1533
7BA	3291	1634	1564
8BA	3313	1633	1532
9BA	3290	1634	1564
10BA	3314	1633	1531

Similarly, N–H (stretch) bands alternate significantly between the odd and even members of the series, for example, the N–H (stretch) band is observed at 3291 cm^–1^ for 5BA and at 3309 cm^–1^ for 6BA. The change in N–H (stretch) is much more dramatic as compared to the C=O band since the hydrogen is more directly influenced by the H-bonding via the N–H bond than the C=O engaged in the H-bonding interaction. In fact, C=O is present in the BA molecule as a more stiff bond, but the hydrogen in N–H is slightly close or slightly further away from the N, depending on the H-bonding network (Table 5).

The amide II band is another characteristic band for *n*BA compounds and is observed at 1563 cm^–1^ for 5BA, shifting to 1533 cm^–1^ for 6BA. The systematic alternation of N–H (stretch) bands and amide II bands between odd and even members, with no significant shift observed between the members of odd or even *n*BA series, is in agreement with the difference in the odd and even H-bonding patterns observed by XRD. The H-bonding network is confining the freedom of the motion of the molecules such that the molecules in odd bisamides feel slightly less constrained than the even molecules since the N–H bands have emerged at lower wave numbers. This systematic shift to higher frequencies for even bisamides compared with odd bisamides can be attributed to stronger H-bonding between N–H and C=O as the H-bond donor and acceptor, respectively, which is in agreement with the observation of XRD, showing a higher packing density for 6BA than 5BA.^44^

## Conclusions

This study provides insights into the effect of odd–even spacer length on the thermal properties and molecular arrangement of model bisamide gelators (*n*BA compounds) in the solid state, aiming at a better understanding of gel systems based on *n*BA compounds. To this end, a homologous series of *n*BA compounds was designed with the (CH2)_*n*_ spacer (*n* = 5–10) between the amide groups and with C17 alkyl tails. Thermal properties as function of the spacer length n were measured by DSC, and subsequently, FTIR spectroscopy, XRD analysis, and molecular modeling were employed to investigate their structural origin. A new analytical model, DSC_N_(T), was developed to reliably obtain the odd–even alternation in melting temperatures, the change in the enthalpy and heat capacity of *n*BA compounds. The not too dramatic difference in thermal properties between the odd and even *n*BA series indicates that the crystal structures of these two groups of bisamides show both differences and similarities.

XRD analysis shows that the studied compounds consist of stacked layers of *n*BA molecules with highly defined layer spacings since they exhibit a series of 00*l* reflections extending up to very high order (*l* = 20). The unit cell of the odd-membered 5BA gelator is pseudo-orthorhombic, whereas that of the even-membered BA6 gelator is triclinic. Their crystalline densities are 1.018 and 1.038 g cm^–3^, respectively. The *c*-axis length increases with increasing spacer length in both odd and even *n*BAs and is close to the length of the gelator molecule, although the molecular axes are tilted somewhat with respect to the *c*-axis. The latter tilting ensures optimal packing of the methyl groups located at each layer surface; the inter-layer interaction is the van der Waals interaction. The layer spacing of the odd-membered 5BA gelator (54.06 Å) is close to the 5BA *c*-axis length (55.58 Å), as described by the pseudo-orthorhombic lattice. The layer spacing of the even-membered 6BA gelator (42.66 Å), however, is much smaller than its *c*-axis length (57.46 Å), and hence much smaller than the 6BA molecular length, due to the triclinic lattice. The very different crystal lattice of odd-membered BAs compared to even-membered BAs translates into a layer spacing close to the molecular length for the odd *n*BAs and with the long molecular axes almost perpendicular to the stacked layers. For the even *n*BAs, the molecular axes are tilted at an angle with respect to the layer normal, which results in a layer spacing markedly smaller than the molecular length. As for the intra-layer structure, the XRD analysis shows that the side-by-side packing of molecules and the H-bonding pattern are similar within the odd/even series but differ markedly between the series. The H-bonding pattern of 5BA can be either sheet-like or bi-directional (or both in different domains). It is different from that of 6BA, which exhibits sheet-like H-bonding very similar to that of Nylon 6,10 α. Moreover, the unit cell parameters of 6BA are remarkably close to those of Nylon 6,10 α (except of course for the *c*-axis length).

The latter observation lends support to the conclusion that the antiparallel amide groups in the even-membered 6BA gelator exhibit a strong structure-directing effect: even though the number of amide groups in 6BA with its C17 alkyl tails is far lower than in Nylon 6,10 α, the 6BA amide groups are still capable of inducing a Nylon 6,10 α-type packing and H-bonding scheme. In contrast with the even-membered 6BA, the unit cell parameters of the odd-membered 5BA are quite close to those of PE (except for the *c*-axis length). The C17 alkyl tails of 5BA have a pseudo-orthorhombic *Z* = 2 *ab* projection cell, similar to the orthorhombic projection cell of PE, and unlike the *Z* = 1 projection cell of 6BA. These differences in the packing of 5BA and 6BA nicely illustrate the different (i.e., lower) structure-directing influences of the parallel amide hydrogen bonding motif in the odd BA series as compared to the antiparallel amide hydrogen bonding motif in the even BA series.

Upon increasing the temperature, melting of the *n*BA compounds is observed in the temperature range of 120–150 °C. The melting temperatures obtained from XRD are in good agreement with the ones obtained from DSC_N_(T). The structural evolution with temperature of the odd-membered 5BA is quite different from that of the even-membered 6BA. The 5BA crystal structure shows a change with temperature to a somewhat higher symmetric structure, which may be a disorder phase. The 6BA crystal structure shows a transition with temperature to a structure with a markedly larger layer spacing than that at ambient temperatures, indicating a reduction of the molecular tilt with respect to the layer normal. Moreover, the high-temperature 6BA structure seems to be quite robust since it remains largely intact up to melting, again indicating the stronger structure-directing effect of antiparallel amide groups, as compared to parallel amide groups. The N–H stretch bands observed using FTIR show an odd–even effect, with the N–H stretch bands of even BAs shifted to higher wavenumbers, pointing to stronger hydrogen bonding between the antiparallel amide groups in the even series than between the parallel amide groups in the odd series. This is in agreement with the XRD results and with the higher melting points of the even BA series.

Summing up, the structural differences observed between the odd and even BA series reflect the different structure-directing effect of parallel versus antiparallel amide hydrogen bonding motifs. These differences, together with the structural evolution with temperature as observed using high-temperature XRD, explain the odd–even effects observed via DSC.

## Supporting Information Available

Full details of the NMR spectra of the whole series of *n*BA compounds, X-ray structure determination, and X-ray crystallographic data (DOCX)
