# Supplementary material for: Molecular Arrangement and Thermal Properties of Bisamide Organogelators in the Solid State
Source: Langmuir. 2022 Nov 23;38(50):15782–95. doi: 10.1021/acs.langmuir.2c02679 (PMC9776524; doi:10.1021/acs.langmuir.2c02679)
Supplement: Supplementary file 1 — la2c02679_si_001.docx [file la2c02679_si_001.docx]

# Supporting information

# Molecular arrangement and thermal properties of bisamide organogelators in the solid state

Authors: Elmira Ghanbari^1^, Aravind Krishnamurthy^1^, Stephen J. Picken^1^, Enno A. Klop^2^, Lars J. Bannenberg^1^, Jan H. van Esch^1,^*

^1^Delft University of Technology, 2629 HZ, Delft, The Netherlands

^2^Teijin Aramid Research and Innovation Centre, P.O. Box 5153, Arnhem, The Netherlands

* Corresponding author’s Email: [j.h.vanesch@tudelft.nl](mailto:j.h.vanesch@tudelft.nl)

Here, more information about the preparation procedure and the analytical data for all nBA compounds are provided.

## Preparation of bisamide gelators

The resulting solid nBA compounds from the synthesis (described in the main text) were ground and recrystallized in ethanol (33 g/L) which yielded yellowish white compounds. The melting points were measured by melting point apparatus and DSC, using the PYRIS analyzer software and with DSC_N_(T) as shown in the Table S1. The melting transition is already starting for the smaller crystals considerably earlier than the temperature that we identify as the melting point using the capillary because the melting point apparatus does not detect the melting point but it detects the point at which a substantial part of the sample has turned into a fluid, which is assessed by visual observation. Therefore, the melting point measured by the capillary is only able to detect the final stage of melting phenomenon while by DSC the onset of melting can be detected as soon as the melting starts. However, the melting point by DSC with the analyzer software is inaccurate due to the asymmetric peak shape for nBA compounds, so we prefer the numbers from our DSC_N_(T) model to deduce the precise melting point of the compounds.

Table S1. Melting point of recrystallized nBA compounds measured by melting point apparatus (MP apparatus) and DSC (the errors are experimental error for the melting points measured by melting apparatus, from DSC measurements and calculated by Pyris software, and for the melting points obtained from DSC_N_(T) model the errors are the fitting residuals).

| nBA compounds | Yield (%) | Melting point (°C)  measured by melting apparatus | Melting point (°C) measured by DSC | Melting point (°C) obtained from the DSC_N_(T) model |
| --- | --- | --- | --- | --- |
| 5BA | 91 | 132±1 | 135±1 | 135.49 ±0.00 |
| 6BA | 89 | 143±1 | 147±1 | 147.46 ±0.00 |
| 7BA | 86 | 132±1 | 135±1 | 135.97 ±0.00 |
| 8BA | 83 | 140±1 | 143±1 | 143.53 ±0.00 |
| 9BA | 94 | 129±1 | 134±1 | 132.50 ±0.00 |
| 10BA | 85 | 138±1 | 141±1 | 139.60 ±0.00 |

Table S2. Fitted parameters and statistical coefficient of the DSC_N_(T) for experimental curves of 6±1 (mg) of odd and even nBA compounds heated at 10 (K.min^-1^) after calibration at the onset for the given weight and rate (the error margins are errors from the nonlinear fitting, excluding errors from the instrument calibration).

| Parameter | 5BA | 6BA | 7BA | 8BA | 9BA | 10BA |
| --- | --- | --- | --- | --- | --- | --- |
| ΔH (J.g^-1^) | 126.18±0.04 | 168.66±0.07 | 140.88±0.09 | 155.12±0.12 | 149.21±0.08 | 172.20±0.06 |
| T_m_^0^ (°C) | 135.49±0.00 | 147.46±0.00 | 135.97±0.00 | 143.53±0.00 | 132.50±0.00 | 142.11±0.00 |
| α (K ^-1^) | 0.39±0.00 | 0.63±0.00 | 0.52±0.00 | 0.31±0.00 | 0.52±0.00 | 0.48±0.00 |
| β (K ^-2^) | 3.01±0.04 | 1. 19±0.02 | 1.50±0.03 | 1.69±0.04 | 2.68±0.05 | 1.40±0.02 |
| ΔC_p,m_ (W.g^-1^.K ^-1^) | -0.44±0.01 | -0.31±0.01 | -0.40±0.01 | -0.28±0.01 | -0.35±0.01 | -0.29±0.01 |
| B (W.g^-1^) | 1.12±0.00 | 1.12±0.01 | 1.04±0.01 | 0.94±0.01 | 1.07±0.01 | 1.19±0.01 |
| C (W.g^-1.^K^-1^) | 0.00±0.00 | 0.00±0.00 | 0.00±0.00 | 0.00±0.00 | 0.00±0.00 | 0.01±0.00 |
| D (W.g^-1^.K^-2^) | 0.00±0.00 | 0.00±0.00 | 0.00±0.00 | 0.00±0.00 | 0.00±0.00 | 0.00±0.00 |
| R^2^ | 0.99 | 0.99 | 0.99 | 0.99 | 0.99 | 0.99 |

Figure S1. NMR spectra of nBA compounds, a) 6BA representing even series and b) 7BA representing odd series c) stacked spectra of odd and even BA compound in DMSO-d_6_ (5 wt%) at 80 (°C), DMSO signal on the spectra (2.5 ppm) and water (3.3 pm), inset of the chemical shift are shown at left.

Figure S2. XRD patterns of synthesized nBA compounds.

Figure S3. Observed 2ϴ positions of the XRD diffraction pattern of 5BA.

Figure S4. Observed 2ϴ positions of the XRD diffraction pattern of 6BA.

## Crystal structure of odd BAs

The peak at 2ϴ=42.20° (Figure 7) is invariant within the odd series, although 5BA indexing shows that it has a very high l index (Table 3). For 5BA the latter peak is indexed as (-2 0 23) and (2 0 22). Figure S5 shows the (-2 0 23) planes, suitably shifted and all atoms in the unit cell are located in the (-2 0 23) planes (to good approximation). Therefore the diffracted X-rays from these planes are all (nearly) in phase, which explains why the (-2 0 23) reflection is relatively strong. A similar figure applies to 7BA, but with the (-2 0 23) planes replaced by the (-2 0 24) planes, since the 7BA spacer has two extra CH_2_ groups, i.e. one extra zig-zag. Likewise, a similar figure also applies for 9BA, with two extra zig-zags and therefore with the (-2 0 23) planes replaced by the (-2 0 25) planes. Clearly, with increasing odd spacer length, the crystal structure remains the same, except for a c-axis length increase by one or two zig-zags, then the indexing of the 42.20° peak will change from (-2 0 23) (5BA) to (-2 0 24) for 7BA and (-2 0 25) for 9BA, in order to accommodate the extra CH_2_ groups, while the spacings of these reflections remains the same. Therefore, the position of the 42.20 °(2θ) peak will be invariant within the odd series.

Figure S5. Crystal structure of 5BA showing the (-2 0 23) planes suitably shifted (which is allowed, since the origin of the unit cell is arbitrary).

Sheet-like H-bonding along the (*010)* planes in each stacked layer of 6BA.

Figure S6. Sheet-like H-bonding between 6BA molecules along the (010) planes in the stacked layers.

Table S3. Fractional atomic coordinates of 5BA 1D-H bonding model.

| Unit cell: a= 8.55 (Å) b=4.36 (Å) c=55.58 (Å)  *α****=***90.00 (°) β=92.09 (°) ϒ=91.92 (°) | | | |
| --- | --- | --- | --- |
| Atom | x | y | z |
| C1 | 0.3123 | 0.5215 | 0.0888 |
| C2 | 0.8116 | 0.0230 | 0.0886 |
| H3 | 0.3114 | 0.4521 | 1.0038 |
| H4 | 0.8120 | -0.0501 | 1.0035 |
| C5 | 0.3972 | 0.5725 | 0.0653 |
| C6 | 0.8978 | 0.0684 | 0.0651 |
| C7 | 0.2983 | 0.4743 | 0.0429 |
| C8 | 0.7983 | -0.0261 | 0.0426 |
| C9 | -0.1145 | 0.0216 | 0.0193 |
| C10 | 0.3844 | 0.5266 | 0.0195 |
| C11 | 0.4122 | 0.6168 | 0.1112 |
| C12 | 0.9126 | 0.1119 | 0.1110 |
| C13 | 0.4460 | 0.6914 | 0.2032 |
| C14 | 0.9467 | 0.1758 | 0.2030 |
| C15 | 0.3275 | 0.5667 | 0.1348 |
| C16 | 0.8260 | 0.0719 | 0.1345 |
| C17 | 0.4655 | 0.7058 | 0.2492 |
| C18 | 0.9660 | 0.1789 | 0.2490 |
| C19 | 0.4884 | 0.6832 | 0.2952 |
| C20 | 0.9879 | 0.1470 | 0.2948 |
| C21 | 0.4284 | 0.6580 | 0.1572 |
| C22 | 0.9289 | 0.1498 | 0.1570 |
| C23 | 0.3436 | 0.6092 | 0.1807 |
| C24 | 0.8410 | 0.1203 | 0.1804 |
| C25 | 0.3796 | 0.6582 | 0.2727 |
| C26 | 0.8782 | 0.1739 | 0.2725 |
| C27 | 0.3606 | 0.6436 | 0.2267 |
| C28 | 0.8578 | 0.1601 | 0.2264 |
| O29 | 0.3280 | 0.4888 | 0.4107 |
| O30 | 0.8345 | 1.0110 | 0.4111 |
| C31 | 0.4596 | 0.4003 | 0.4079 |
| C32 | 0.9610 | 0.9010 | 0.4079 |
| C33 | 0.5655 | 0.2267 | 0.4699 |
| C34 | 1.0648 | 0.7298 | 0.4698 |
| C35 | 0.4360 | 0.5494 | 0.3645 |
| C36 | 0.9373 | 0.0487 | 0.3644 |
| C37 | 0.4037 | 0.6336 | 0.3188 |
| C38 | 0.9043 | 0.1418 | 0.3187 |
| C39 | 0.4683 | 0.1446 | 0.4470 |
| C40 | 0.9659 | -0.3531 | 0.4471 |
| N41 | 0.5398 | 0.2462 | 0.4249 |
| N42 | 1.0373 | -0.2623 | 0.4247 |
| C43 | 0.5170 | 0.6026 | 0.3406 |
| C44 | 1.0153 | 0.0683 | 0.3400 |
| C45 | 0.5502 | 0.4549 | 0.3848 |
| C46 | 1.0488 | -0.0677 | 0.3843 |
| C47 | 0.4826 | 0.1294 | 0.4928 |
| C48 | 0.9823 | 0.6349 | 0.4929 |
| O49 | 0.3711 | 0.4562 | 0.5755 |
| O50 | 0.8714 | -0.0423 | 0.5755 |
| C51 | -0.0001 | -0.1514 | 0.5785 |
| C52 | 0.4996 | 0.3474 | 0.5785 |
| C53 | 0.0753 | 0.7110 | 0.5163 |
| C54 | 0.5748 | 0.2075 | 0.5163 |
| C55 | 0.0006 | 0.9949 | 0.6221 |
| C56 | 0.5006 | 0.4948 | 0.6220 |
| C57 | -0.0047 | 1.0913 | 0.6679 |
| C58 | 0.4953 | 0.5916 | 0.6679 |
| C59 | 0.4868 | 0.1072 | 0.5387 |
| C60 | 0.9873 | 0.6100 | 0.5386 |
| N61 | 0.0683 | 0.6906 | 0.5613 |
| N62 | 0.5679 | 0.1888 | 0.5613 |
| C63 | 0.0899 | 0.9938 | 0.6465 |
| C64 | 0.5899 | 0.4941 | 0.6464 |
| C65 | 0.1000 | 0.8740 | 0.6020 |
| C66 | 0.5998 | 0.3736 | 0.6020 |
| C67 | 0.1637 | 1.0201 | 0.8747 |
| C68 | 0.6638 | 0.5201 | 0.8747 |
| C69 | 0.1138 | 1.0722 | 0.7831 |
| C70 | 0.6137 | 0.5727 | 0.7831 |
| C71 | 0.0601 | 1.1080 | 0.8528 |
| C72 | 0.5602 | 0.6080 | 0.8528 |
| C73 | 0.0965 | 1.0756 | 0.7374 |
| C74 | 0.5964 | 0.5763 | 0.7374 |
| C75 | 0.0881 | 1.0540 | 0.6918 |
| C76 | 0.5881 | 0.5545 | 0.6917 |
| C77 | 0.1372 | 1.0519 | 0.8289 |
| C78 | 0.6372 | 0.5521 | 0.8289 |
| C79 | 0.0349 | 1.1360 | 0.8068 |
| C80 | 0.5348 | 0.6362 | 0.8068 |
| C81 | 0.0000 | 1.1377 | 0.7142 |
| C82 | 0.4999 | 0.6381 | 0.7142 |
| C83 | 0.0140 | 1.1487 | 0.7606 |
| C84 | 0.5138 | 0.6491 | 0.7606 |
| C85 | 0.0868 | 1.0699 | 0.8987 |
| C86 | 0.5870 | 0.5698 | 0.8987 |
| H87 | 0.1612 | 0.9599 | 0.9832 |
| H88 | 0.6616 | 0.4590 | 0.9832 |
| C89 | 0.1908 | 0.9801 | 0.9205 |
| C90 | 0.6910 | 0.4799 | 0.9205 |
| C91 | 0.1138 | 1.0241 | 0.9446 |
| C92 | 0.6141 | 0.5237 | 0.9446 |
| C93 | 0.2192 | 0.9316 | 0.9660 |
| C94 | 0.7195 | 0.4308 | 0.9660 |
| H95 | 0.2036 | 0.6515 | 0.0883 |
| H96 | 0.7054 | 0.1601 | 0.0880 |
| H97 | 0.2762 | 0.2769 | 0.0903 |
| H98 | 0.7715 | 0.7806 | 0.0900 |
| H99 | -0.0639 | 0.3117 | 0.0635 |
| H100 | 0.4324 | 0.8175 | 0.0637 |
| H101 | 0.0052 | 0.9347 | 0.0658 |
| H102 | 0.5064 | 0.4442 | 0.0659 |
| H103 | 0.2634 | 0.2292 | 0.0444 |
| H104 | 0.7607 | 0.7302 | 0.0442 |
| H105 | 0.1890 | 0.6020 | 0.0422 |
| H106 | 0.6905 | 0.1065 | 0.0418 |
| H107 | -0.0078 | 0.8870 | 0.0195 |
| H108 | 0.4927 | 0.3969 | 0.0196 |
| H109 | -0.0796 | 0.2649 | 0.0170 |
| H110 | 0.4166 | 0.7712 | 0.0172 |
| H111 | 0.0177 | 0.9715 | 0.1117 |
| H112 | 0.5206 | 0.4861 | 0.1118 |
| H113 | -0.0453 | 0.3530 | 0.1094 |
| H114 | 0.4487 | 0.8612 | 0.1097 |
| H115 | 0.0384 | 1.0033 | 0.2035 |
| H116 | 0.5508 | 0.5502 | 0.2035 |
| H117 | 0.0077 | 0.4021 | 0.2017 |
| H118 | 0.4884 | 0.9328 | 0.2020 |
| H119 | 0.2198 | 0.6994 | 0.1343 |
| H120 | 0.7230 | 0.2182 | 0.1340 |
| H121 | 0.2899 | 0.3227 | 0.1362 |
| H122 | 0.7805 | 0.8326 | 0.1358 |
| H123 | 0.0412 | 0.3892 | 0.2484 |
| H124 | 0.5152 | 0.9426 | 0.2487 |
| H125 | 0.0449 | 0.9837 | 0.2489 |
| H126 | 0.5654 | 0.5520 | 0.2491 |
| H127 | 0.0529 | 0.9337 | 0.2932 |
| H128 | 0.5793 | 0.5107 | 0.2939 |
| H129 | 0.0759 | 0.3380 | 0.2950 |
| H130 | 0.5499 | 0.9098 | 0.2957 |
| H131 | 0.0293 | 0.9970 | 0.1577 |
| H132 | 0.5356 | 0.5237 | 0.1576 |
| H133 | -0.0218 | 0.3861 | 0.1554 |
| H134 | 0.4669 | 0.9014 | 0.1557 |
| H135 | 0.3027 | 0.3669 | 0.1819 |
| H136 | 0.7853 | 0.8888 | 0.1815 |
| H137 | 0.2379 | 0.7478 | 0.1805 |
| H138 | 0.7453 | 0.2841 | 0.1801 |
| H139 | 0.2866 | 0.8267 | 0.2738 |
| H140 | 0.8100 | 0.3833 | 0.2738 |
| H141 | 0.3206 | 0.4289 | 0.2724 |
| H142 | 0.7930 | 0.9780 | 0.2723 |
| H143 | 0.2593 | 0.7936 | 0.2268 |
| H144 | 0.7744 | 0.3476 | 0.2266 |
| H145 | 0.3130 | 0.4052 | 0.2274 |
| H146 | 0.7874 | 0.9434 | 0.2269 |
| H147 | 0.0911 | -0.0208 | 0.4701 |
| H148 | 0.5906 | 0.4765 | 0.4703 |
| H149 | 0.1782 | 0.6176 | 0.4692 |
| H150 | 0.6797 | 0.1169 | 0.4694 |
| H151 | 0.3764 | 0.7595 | 0.3694 |
| H152 | 0.8945 | 0.2758 | 0.3692 |
| H153 | 0.3450 | 0.3654 | 0.3619 |
| H154 | 0.8344 | 0.8895 | 0.3627 |
| H155 | 0.3251 | 0.8247 | 0.3216 |
| H156 | 0.8504 | 0.3647 | 0.3216 |
| H157 | 0.3289 | 0.4227 | 0.3173 |
| H158 | 0.8079 | 0.9669 | 0.3178 |
| H159 | 0.4455 | -0.1063 | 0.4464 |
| H160 | 0.9403 | 0.3968 | 0.4469 |
| H161 | 0.3528 | 0.2472 | 0.4481 |
| H162 | 0.8516 | -0.2452 | 0.4481 |
| H163 | 0.1393 | -0.3581 | 0.4207 |
| H164 | 0.6457 | 0.1650 | 0.4211 |
| H165 | 0.1123 | 0.2417 | 0.3411 |
| H166 | 0.5946 | 0.8093 | 0.3422 |
| H167 | 0.0684 | 0.8461 | 0.3365 |
| H168 | 0.5929 | 0.4081 | 0.3373 |
| H169 | 0.1506 | 0.0919 | 0.3869 |
| H170 | 0.6415 | 0.6358 | 0.3881 |
| H171 | 0.0931 | 0.7096 | 0.3787 |
| H172 | 0.6088 | 0.2463 | 0.3792 |
| H173 | 0.3683 | 0.2399 | 0.4930 |
| H174 | 0.8686 | -0.2525 | 0.4931 |
| H175 | 0.4567 | -0.1204 | 0.4922 |
| H176 | 0.9552 | 0.3855 | 0.4922 |
| H177 | 0.1892 | 0.5996 | 0.5163 |
| H178 | 0.6890 | 0.0974 | 0.5164 |
| H179 | 0.1013 | -0.0398 | 0.5173 |
| H180 | 0.6001 | 0.4570 | 0.5172 |
| H181 | 0.3938 | 0.3473 | 0.6233 |
| H182 | 0.8938 | 0.8475 | 0.6233 |
| H183 | 0.4627 | 0.7284 | 0.6177 |
| H184 | 0.9628 | 0.2286 | 0.6178 |
| H185 | -0.0392 | 0.3315 | 0.6656 |
| H186 | 0.4607 | 0.8317 | 0.6656 |
| H187 | 0.3859 | 0.4506 | 0.6683 |
| H188 | 0.8858 | 0.9505 | 0.6683 |
| H189 | 0.3710 | 0.2108 | 0.5381 |
| H190 | 0.8715 | -0.2861 | 0.5381 |
| H191 | 0.4637 | -0.1436 | 0.5380 |
| H192 | 0.9640 | 0.3593 | 0.5379 |
| H193 | 0.1728 | 0.5960 | 0.5651 |
| H194 | 0.6725 | 0.0948 | 0.5651 |
| H195 | 0.1311 | 0.7597 | 0.6499 |
| H196 | 0.6312 | 0.2601 | 0.6498 |
| H197 | 0.1958 | 0.1443 | 0.6455 |
| H198 | 0.6957 | 0.6447 | 0.6455 |
| H199 | 0.1439 | 0.6480 | 0.6074 |
| H200 | 0.6440 | 0.1480 | 0.6074 |
| H201 | 0.2026 | 0.0284 | 0.5994 |
| H202 | 0.7024 | 0.5283 | 0.5993 |
| H203 | 0.1939 | 0.7767 | 0.8731 |
| H204 | 0.6940 | 0.2768 | 0.8731 |
| H205 | 0.2753 | 0.1547 | 0.8745 |
| H206 | 0.7753 | 0.6548 | 0.8744 |
| H207 | 0.2260 | 0.2043 | 0.7828 |
| H208 | 0.7259 | 0.7051 | 0.7828 |
| H209 | 0.1430 | 0.8276 | 0.7825 |
| H210 | 0.6431 | 0.3281 | 0.7825 |
| H211 | 0.4481 | 0.4752 | 0.8532 |
| H212 | 0.9481 | 0.9752 | 0.8532 |
| H213 | 0.5312 | 0.8521 | 0.8542 |
| H214 | 1.0311 | 0.3520 | 0.8542 |
| H215 | 0.1274 | 0.8316 | 0.7377 |
| H216 | 0.6275 | 0.3324 | 0.7376 |
| H217 | 0.2081 | 0.2098 | 0.7371 |
| H218 | 0.7080 | 0.7108 | 0.7371 |
| H219 | 0.1232 | 0.8130 | 0.6933 |
| H220 | 0.6233 | 0.3137 | 0.6933 |
| H221 | 0.1976 | 0.1949 | 0.6913 |
| H222 | 0.6975 | 0.6957 | 0.6913 |
| H223 | 0.2492 | 0.1848 | 0.8286 |
| H224 | 0.7492 | 0.6851 | 0.8286 |
| H225 | 0.1666 | 0.8078 | 0.8277 |
| H226 | 0.6666 | 0.3080 | 0.8277 |
| H227 | 0.5065 | 0.8810 | 0.8077 |
| H228 | 1.0067 | 0.3809 | 0.8077 |
| H229 | 0.4224 | 0.5049 | 0.8072 |
| H230 | 0.9224 | 1.0049 | 0.8072 |
| H231 | 0.3884 | 0.5036 | 0.7144 |
| H232 | 0.8884 | 1.0035 | 0.7145 |
| H233 | -0.0304 | 0.3815 | 0.7135 |
| H234 | 0.4693 | 0.8819 | 0.7134 |
| H235 | 0.4014 | 0.5178 | 0.7609 |
| H236 | 0.9014 | 1.0177 | 0.7609 |
| H237 | 0.4853 | 0.8940 | 0.7608 |
| H238 | 0.9855 | 0.3937 | 0.7608 |
| H239 | 0.5568 | 0.8129 | 0.9004 |
| H240 | 1.0566 | 0.3130 | 0.9004 |
| H241 | 0.4755 | 0.4349 | 0.8989 |
| H242 | 0.9754 | 0.9349 | 0.8989 |
| H243 | 0.2221 | 0.7377 | 0.9187 |
| H244 | 0.7223 | 0.2375 | 0.9186 |
| H245 | 0.3017 | 0.1169 | 0.9204 |
| H246 | 0.8020 | 0.6166 | 0.9204 |
| H247 | 0.5829 | 0.7657 | 0.9466 |
| H248 | 1.0826 | 0.2661 | 0.9466 |
| H249 | 0.5033 | 0.3867 | 0.9447 |
| H250 | 1.0031 | 0.8870 | 0.9447 |
| H251 | 0.3287 | 0.0721 | 0.9669 |
| H252 | 0.8290 | 0.5713 | 0.9668 |
| H253 | 0.2505 | 0.6895 | 0.9645 |
| H254 | 0.7508 | 0.1888 | 0.9645 |

Table S4. Fractional atomic coordinates of 5BA 2D-H bonding model.

| Unit cell: a= 8.55 (Å) b=4.36 (Å) c=55.58 (Å)  *α****=***90.00 (°) β=92.09 (°) ϒ=91.92 (°) | | | |
| --- | --- | --- | --- |
| C1 | 0.3088 | 0.4120 | 0.0810 |
| C2 | 0.8088 | -0.0880 | 0.0810 |
| H3 | 0.3249 | 0.4140 | 0.9960 |
| H4 | 0.8249 | -0.0860 | 0.9960 |
| C5 | 0.3987 | 0.4820 | 0.0582 |
| C6 | 0.8987 | -0.0180 | 0.0582 |
| C7 | 0.3044 | 0.4002 | 0.0350 |
| C8 | 0.8044 | -0.0998 | 0.0350 |
| C9 | -0.1045 | -0.0289 | 0.0124 |
| C10 | 0.3955 | 0.4711 | 0.0124 |
| C11 | 0.4020 | 0.4967 | 0.1042 |
| C12 | 0.9020 | -0.0033 | 0.1042 |
| C13 | 0.4059 | 0.5107 | 0.1962 |
| C14 | 0.9059 | 0.0107 | 0.1962 |
| C15 | 0.3113 | 0.4248 | 0.1269 |
| C16 | 0.8113 | -0.0752 | 0.1269 |
| C17 | 0.4118 | 0.5040 | 0.2421 |
| C18 | 0.9118 | 0.0040 | 0.2421 |
| C19 | 0.4248 | 0.4907 | 0.2879 |
| C20 | 0.9248 | -0.0093 | 0.2879 |
| C21 | 0.4037 | 0.5079 | 0.1503 |
| C22 | 0.9037 | 0.0079 | 0.1503 |
| C23 | 0.3130 | 0.4314 | 0.1729 |
| C24 | 0.8130 | -0.0686 | 0.1729 |
| C25 | 0.3261 | 0.4165 | 0.2650 |
| C26 | 0.8261 | -0.0835 | 0.2650 |
| C27 | 0.3168 | 0.4285 | 0.2189 |
| C28 | 0.8168 | -0.0715 | 0.2189 |
| O29 | 0.3021 | 0.2166 | 0.4041 |
| O30 | 0.8021 | 0.7166 | 0.4041 |
| C31 | 0.4307 | 0.3404 | 0.4022 |
| C32 | 0.9308 | 0.8405 | 0.4022 |
| C33 | 0.5890 | 0.3548 | 0.4661 |
| C34 | 1.0890 | 0.8548 | 0.4661 |
| C35 | 0.3822 | 0.3765 | 0.3574 |
| C36 | 0.8822 | -0.1235 | 0.3574 |
| C37 | 0.3454 | 0.3985 | 0.3112 |
| C38 | 0.8453 | -0.1016 | 0.3112 |
| C39 | 0.4792 | 0.2694 | 0.4446 |
| C40 | 0.9792 | -0.2306 | 0.4446 |
| N41 | 0.5292 | 0.3850 | 0.4214 |
| N42 | 1.0292 | -0.1151 | 0.4214 |
| C43 | 0.4503 | 0.4748 | 0.3334 |
| C44 | 0.9503 | -0.0252 | 0.3334 |
| C45 | 0.4967 | 0.4580 | 0.3784 |
| C46 | 0.9967 | -0.0420 | 0.3784 |
| C47 | 0.5189 | 0.2497 | 0.4898 |
| C48 | 1.0189 | 0.7497 | 0.4898 |
| O49 | 0.4182 | 0.5842 | 0.5715 |
| O50 | 0.9182 | 0.0842 | 0.5715 |
| C51 | 0.0467 | -0.0239 | 0.5747 |
| C52 | 0.5467 | 0.4761 | 0.5747 |
| C53 | 0.1166 | 0.8322 | 0.5127 |
| C54 | 0.6166 | 0.3322 | 0.5127 |
| C55 | 0.0439 | 1.1312 | 0.6180 |
| C56 | 0.5439 | 0.6312 | 0.6180 |
| C57 | 0.0318 | 1.2334 | 0.6636 |
| C58 | 0.5317 | 0.7334 | 0.6636 |
| C59 | 0.5343 | 0.2247 | 0.5354 |
| C60 | 1.0343 | 0.7248 | 0.5354 |
| N61 | 0.1155 | 0.8133 | 0.5579 |
| N62 | 0.6155 | 0.3133 | 0.5579 |
| C63 | 0.1301 | 1.1351 | 0.6426 |
| C64 | 0.6301 | 0.6351 | 0.6426 |
| C65 | 0.1456 | 1.0079 | 0.5983 |
| C66 | 0.6456 | 0.5079 | 0.5983 |
| C67 | 0.1699 | 1.0803 | 0.8701 |
| C68 | 0.6699 | 0.5802 | 0.8701 |
| C69 | 0.1295 | 1.1842 | 0.7790 |
| C70 | 0.6295 | 0.6842 | 0.7790 |
| C71 | 0.0665 | 1.1771 | 0.8485 |
| C72 | 0.5665 | 0.6771 | 0.8485 |
| C73 | 0.1201 | 1.2064 | 0.7334 |
| C74 | 0.6201 | 0.7064 | 0.7334 |
| C75 | 0.1203 | 1.1955 | 0.6878 |
| C76 | 0.6203 | 0.6955 | 0.6878 |
| C77 | 0.1468 | 1.1401 | 0.8246 |
| C78 | 0.6468 | 0.6401 | 0.8246 |
| C79 | 0.0464 | 1.2326 | 0.8026 |
| C80 | 0.5464 | 0.7326 | 0.8026 |
| C81 | 0.0278 | 1.2748 | 0.7099 |
| C82 | 0.5278 | 0.7748 | 0.7099 |
| C83 | 0.0330 | 1.2691 | 0.7563 |
| C84 | 0.5330 | 0.7691 | 0.7563 |
| C85 | 0.0917 | 1.1091 | 0.8943 |
| C86 | 0.5917 | 0.6091 | 0.8943 |
| H87 | 0.1723 | 0.9432 | 0.9782 |
| H88 | 0.6723 | 0.4432 | 0.9782 |
| C89 | 0.1968 | 1.0079 | 0.9156 |
| C90 | 0.6968 | 0.5079 | 0.9156 |
| C91 | 0.1209 | 1.0320 | 0.9400 |
| C92 | 0.6209 | 0.5321 | 0.9400 |
| C93 | 0.2283 | 0.9255 | 0.9607 |
| C94 | 0.7283 | 0.4255 | 0.9607 |
| H95 | 0.1987 | 0.5375 | 0.0803 |
| H96 | 0.6987 | 0.0375 | 0.0803 |
| H97 | 0.2750 | 0.1655 | 0.0814 |
| H98 | 0.7750 | 0.6655 | 0.0814 |
| H99 | -0.0666 | 0.2282 | 0.0579 |
| H100 | 0.4334 | 0.7282 | 0.0579 |
| H101 | 0.0083 | 0.8548 | 0.0588 |
| H102 | 0.5083 | 0.3548 | 0.0588 |
| H103 | 0.2696 | 0.1542 | 0.0352 |
| H104 | 0.7696 | 0.6542 | 0.0352 |
| H105 | 0.1950 | 0.5276 | 0.0344 |
| H106 | 0.6950 | 0.0276 | 0.0344 |
| H107 | 0.0026 | 0.8380 | 0.0122 |
| H108 | 0.5026 | 0.3380 | 0.0122 |
| H109 | -0.0699 | 0.2163 | 0.0116 |
| H110 | 0.4301 | 0.7163 | 0.0116 |
| H111 | 0.0123 | 0.8720 | 0.1050 |
| H112 | 0.5123 | 0.3720 | 0.1050 |
| H113 | -0.0647 | 0.2435 | 0.1040 |
| H114 | 0.4354 | 0.7435 | 0.1040 |
| H115 | 0.0170 | 0.8886 | 0.1966 |
| H116 | 0.5170 | 0.3886 | 0.1966 |
| H117 | -0.0622 | 0.2582 | 0.1965 |
| H118 | 0.4378 | 0.7582 | 0.1965 |
| H119 | 0.2009 | 0.5494 | 0.1262 |
| H120 | 0.7009 | 0.0494 | 0.1262 |
| H121 | 0.2780 | 0.1779 | 0.1271 |
| H122 | 0.7780 | 0.6779 | 0.1271 |
| H123 | -0.0571 | 0.2518 | 0.2426 |
| H124 | 0.4429 | 0.7518 | 0.2426 |
| H125 | 0.0233 | 0.8834 | 0.2420 |
| H126 | 0.5233 | 0.3834 | 0.2420 |
| H127 | 0.0369 | 0.8730 | 0.2871 |
| H128 | 0.5369 | 0.3730 | 0.2871 |
| H129 | -0.0454 | 0.2390 | 0.2884 |
| H130 | 0.4546 | 0.7390 | 0.2884 |
| H131 | 0.0145 | 0.8848 | 0.1509 |
| H132 | 0.5145 | 0.3848 | 0.1509 |
| H133 | -0.0639 | 0.2552 | 0.1502 |
| H134 | 0.4361 | 0.7552 | 0.1502 |
| H135 | 0.2799 | 0.1844 | 0.1728 |
| H136 | 0.7799 | 0.6844 | 0.1728 |
| H137 | 0.2026 | 0.5557 | 0.1723 |
| H138 | 0.7026 | 0.0557 | 0.1723 |
| H139 | 0.2150 | 0.5380 | 0.2654 |
| H140 | 0.7150 | 0.0380 | 0.2654 |
| H141 | 0.2946 | 0.1689 | 0.2645 |
| H142 | 0.7946 | 0.6689 | 0.2645 |
| H143 | 0.2061 | 0.5519 | 0.2187 |
| H144 | 0.7061 | 0.0519 | 0.2187 |
| H145 | 0.2842 | 0.1813 | 0.2186 |
| H146 | 0.7842 | 0.6813 | 0.2186 |
| H147 | 0.1107 | 0.1055 | 0.4665 |
| H148 | 0.6107 | 0.6055 | 0.4665 |
| H149 | 0.2036 | 0.7514 | 0.4639 |
| H150 | 0.7036 | 0.2514 | 0.4639 |
| H151 | 0.2700 | 0.4886 | 0.3598 |
| H152 | 0.7700 | -0.0114 | 0.3598 |
| H153 | 0.3570 | 0.1266 | 0.3571 |
| H154 | 0.8570 | 0.6266 | 0.3571 |
| H155 | 0.2336 | 0.5162 | 0.3123 |
| H156 | 0.7336 | 0.0162 | 0.3123 |
| H157 | 0.3159 | 0.1500 | 0.3108 |
| H158 | 0.8159 | 0.6500 | 0.3108 |
| H159 | 0.4638 | 0.0169 | 0.4438 |
| H160 | 0.9638 | 0.5169 | 0.4438 |
| H161 | 0.3627 | 0.3598 | 0.4479 |
| H162 | 0.8627 | -0.1403 | 0.4479 |
| H163 | 0.1374 | -0.0199 | 0.4195 |
| H164 | 0.6374 | 0.4801 | 0.4195 |
| H165 | -0.0230 | 0.2243 | 0.3339 |
| H166 | 0.4770 | 0.7243 | 0.3339 |
| H167 | 0.0634 | 0.8634 | 0.3316 |
| H168 | 0.5634 | 0.3634 | 0.3316 |
| H169 | 0.0174 | 0.2090 | 0.3792 |
| H170 | 0.5174 | 0.7090 | 0.3792 |
| H171 | 0.1099 | 0.8537 | 0.3753 |
| H172 | 0.6099 | 0.3538 | 0.3753 |
| H173 | 0.4028 | 0.3512 | 0.4912 |
| H174 | 0.9028 | -0.1489 | 0.4912 |
| H175 | 0.4974 | -0.0014 | 0.4892 |
| H176 | 0.9974 | 0.4986 | 0.4892 |
| H177 | 0.2323 | 0.7288 | 0.5121 |
| H178 | 0.7323 | 0.2288 | 0.5121 |
| H179 | 0.1382 | 0.0827 | 0.5136 |
| H180 | 0.6382 | 0.5827 | 0.5136 |
| H181 | 0.4373 | 0.4829 | 0.6191 |
| H182 | 0.9373 | 0.9829 | 0.6191 |
| H183 | 0.5059 | 0.8638 | 0.6134 |
| H184 | 1.0060 | 0.3638 | 0.6134 |
| H185 | -0.0024 | 0.4736 | 0.6612 |
| H186 | 0.4976 | 0.9736 | 0.6612 |
| H187 | 0.4222 | 0.5925 | 0.6636 |
| H188 | 0.9222 | 1.0925 | 0.6636 |
| H189 | 0.4159 | 0.3171 | 0.5351 |
| H190 | 0.9159 | -0.1829 | 0.5351 |
| H191 | 0.5171 | -0.0274 | 0.5348 |
| H192 | 1.0171 | 0.4726 | 0.5348 |
| H193 | 0.2203 | 0.7209 | 0.5618 |
| H194 | 0.7203 | 0.2209 | 0.5618 |
| H195 | 0.1718 | 0.9022 | 0.6464 |
| H196 | 0.6718 | 0.4022 | 0.6464 |
| H197 | 0.2357 | 0.2869 | 0.6419 |
| H198 | 0.7356 | 0.7869 | 0.6419 |
| H199 | 0.1903 | 0.7840 | 0.6041 |
| H200 | 0.6903 | 0.2840 | 0.6041 |
| H201 | 0.2478 | 0.1634 | 0.5957 |
| H202 | 0.7478 | 0.6634 | 0.5957 |
| H203 | 0.2033 | 0.8402 | 0.8676 |
| H204 | 0.7033 | 0.3402 | 0.8676 |
| H205 | 0.2798 | 0.2206 | 0.8705 |
| H206 | 0.7798 | 0.7206 | 0.8706 |
| H207 | 0.2409 | 0.3196 | 0.7795 |
| H208 | 0.7409 | 0.8196 | 0.7795 |
| H209 | 0.1607 | 0.9411 | 0.7777 |
| H210 | 0.6607 | 0.4411 | 0.7777 |
| H211 | 0.4561 | 0.5386 | 0.8481 |
| H212 | 0.9561 | 1.0386 | 0.8481 |
| H213 | 0.5343 | 0.9182 | 0.8508 |
| H214 | 1.0343 | 0.4182 | 0.8508 |
| H215 | 0.1520 | 0.9629 | 0.7333 |
| H216 | 0.6520 | 0.4629 | 0.7333 |
| H217 | 0.2313 | 0.3425 | 0.7338 |
| H218 | 0.7313 | 0.8425 | 0.7338 |
| H219 | 0.1560 | 0.9549 | 0.6894 |
| H220 | 0.6560 | 0.4549 | 0.6894 |
| H221 | 0.2296 | 0.3378 | 0.6879 |
| H222 | 0.7296 | 0.8378 | 0.6879 |
| H223 | 0.2574 | 0.2783 | 0.8250 |
| H224 | 0.7574 | 0.7783 | 0.8250 |
| H225 | 0.1793 | 0.8986 | 0.8226 |
| H226 | 0.6793 | 0.3986 | 0.8226 |
| H227 | 0.5157 | 0.9754 | 0.8042 |
| H228 | 1.0157 | 0.4754 | 0.8042 |
| H229 | 0.4350 | 0.5972 | 0.8021 |
| H230 | 0.9350 | 1.0972 | 0.8021 |
| H231 | 0.4160 | 0.6407 | 0.7095 |
| H232 | 0.9160 | 1.1407 | 0.7095 |
| H233 | -0.0023 | 0.5188 | 0.7094 |
| H234 | 0.4977 | 1.0188 | 0.7094 |
| H235 | 0.4208 | 0.6363 | 0.7559 |
| H236 | 0.9208 | 1.1362 | 0.7559 |
| H237 | 0.5036 | 1.0134 | 0.7571 |
| H238 | 1.0036 | 0.5134 | 0.7571 |
| H239 | 0.5589 | 0.8491 | 0.8970 |
| H240 | 1.0589 | 0.3491 | 0.8970 |
| H241 | 0.4817 | 0.4693 | 0.8939 |
| H242 | 0.9817 | 0.9693 | 0.8939 |
| H243 | 0.2299 | 0.7684 | 0.9127 |
| H244 | 0.7299 | 0.2684 | 0.9127 |
| H245 | 0.3067 | 0.1481 | 0.9160 |
| H246 | 0.8067 | 0.6481 | 0.9160 |
| H247 | 0.5889 | 0.7715 | 0.9431 |
| H248 | 1.0889 | 0.2715 | 0.9431 |
| H249 | 0.5107 | 0.3930 | 0.9397 |
| H250 | 1.0107 | 0.8930 | 0.9397 |
| H251 | 0.3381 | 0.0647 | 0.9618 |
| H252 | 0.8381 | 0.5647 | 0.9618 |
| H253 | 0.2588 | 0.6846 | 0.9582 |
| H254 | 0.7588 | 0.1846 | 0.9582 |

Table S5. Fractional atomic coordinates of 6BA.

| Unit cell: a= 5.02 (Å) b=5.29 (Å) c=57.46 (Å)  *α****=***48.65 (°) β=76.80 (°) ϒ=65.17 (°) | | | |
| --- | --- | --- | --- |
| Atom | x | y | z |
| C1 | -0.2030 | 0.0149 | 0.1029 |
| C2 | -0.3744 | 0.0462 | 0.1054 |
| C3 | -0.1795 | -0.0056 | 0.1074 |
| C4 | -0.3480 | 0.0278 | 0.1099 |
| C5 | -0.1521 | -0.0245 | 0.1119 |
| C6 | -0.3210 | 0.0137 | 0.1143 |
| C7 | -0.1252 | -0.0385 | 0.1164 |
| C8 | -0.2950 | 0.0027 | 0.1188 |
| C9 | -0.0996 | -0.0492 | 0.1209 |
| C10 | -0.2698 | -0.0064 | 0.1232 |
| C11 | -0.0741 | -0.0575 | 0.1253 |
| C12 | -0.2433 | -0.0148 | 0.1277 |
| C13 | -0.0465 | -0.0640 | 0.1297 |
| C14 | -0.2139 | -0.0223 | 0.1321 |
| C15 | -0.0160 | -0.0671 | 0.1341 |
| C16 | -0.1810 | -0.0270 | 0.1365 |
| C17 | 0.0194 | -0.0654 | 0.1385 |
| C18 | -0.1632 | -0.0255 | 0.1408 |
| C19 | -0.1643 | 0.0238 | 0.1447 |
| C20 | 0.0440 | -0.0212 | 0.1466 |
| C21 | -0.1065 | 0.0212 | 0.1491 |
| O1 | -0.4183 | -0.0038 | 0.1412 |
| N1 | -0.0135 | -0.0243 | 0.1425 |
| H1 | -0.0238 | -0.2517 | 0.1041 |
| H2 | -0.3461 | 0.0482 | 0.1015 |
| H3 | -0.1046 | 0.2235 | 0.1014 |
| H4 | -0.4793 | -0.1568 | 0.1068 |
| H5 | -0.5540 | 0.3141 | 0.1042 |
| H6 | -0.0004 | -0.2740 | 0.1086 |
| H7 | -0.0735 | 0.1964 | 0.1060 |
| H8 | -0.4545 | -0.1737 | 0.1113 |
| H9 | -0.5267 | 0.2966 | 0.1087 |
| H10 | 0.0255 | -0.2942 | 0.1132 |
| H11 | -0.0438 | 0.1752 | 0.1105 |
| H12 | -0.4981 | 0.2839 | 0.1131 |
| H13 | -0.4297 | -0.1855 | 0.1158 |
| H14 | 0.0512 | -0.3092 | 0.1177 |
| H15 | -0.0154 | 0.1596 | 0.1150 |
| H16 | -0.4049 | -0.1952 | 0.1203 |
| H17 | -0.4713 | 0.2736 | 0.1176 |
| H18 | 0.0764 | -0.3203 | 0.1221 |
| H19 | 0.0108 | 0.1482 | 0.1194 |
| H20 | -0.4458 | 0.2647 | 0.1220 |
| H21 | -0.3799 | -0.2040 | 0.1247 |
| H22 | 0.0359 | 0.1403 | 0.1238 |
| H23 | 0.1020 | -0.3284 | 0.1266 |
| H24 | -0.4201 | 0.2556 | 0.1264 |
| H25 | -0.3525 | -0.2133 | 0.1292 |
| H26 | 0.1309 | -0.3340 | 0.1310 |
| H27 | 0.0619 | 0.1353 | 0.1282 |
| H28 | -0.3922 | 0.2470 | 0.1309 |
| H29 | -0.3210 | -0.2229 | 0.1336 |
| H30 | 0.0898 | 0.1349 | 0.1326 |
| H31 | 0.1634 | -0.3355 | 0.1353 |
| H32 | -0.3619 | 0.2401 | 0.1353 |
| H33 | -0.2821 | -0.2334 | 0.1381 |
| H34 | 0.1201 | 0.1411 | 0.1369 |
| H35 | 0.1998 | -0.3329 | 0.1397 |
| H36 | 0.1923 | -0.0213 | 0.1420 |
| H37 | -0.2713 | -0.1762 | 0.1463 |
| H38 | -0.3414 | 0.2940 | 0.1435 |
| H39 | 0.1503 | 0.1794 | 0.1451 |
| H40 | 0.2221 | -0.2899 | 0.1478 |
| H41 | -0.2852 | 0.2900 | 0.1479 |
| H42 | -0.2116 | -0.1805 | 0.1507 |
